# Supplementary material for: Dynamics of ranking
Source: Nat Commun. 2022 Mar 28;13:1646. doi: 10.1038/s41467-022-29256-x (PMC8960905; doi:10.1038/s41467-022-29256-x)
Supplement: Supplementary file 1 — Supplementary Information [file 41467_2022_29256_MOESM1_ESM.pdf]

Supplementary Information for

Dynamics of ranking

G. Iñiguez, C. Pineda, C. Gershenson, and A.-L. Barabási

Contents

S1 Summary of notation **2**

S2 Data description **2**

S2.1 Society . . . 2
S2.2 Languages . . . 4
S2.3 Economics . . . 4
S2.4 Infrastructure . . . 4
S2.5 Nature . . . 5
S2.6 Sports . . . 5
S2.7 Acknowledgments and data availability . . . 6

S3 Rank measures **6**

S3.1 Fitness . . . 7
S3.2 Rank dynamics . . . 7
S3.3 Rank flux . . . 8
S3.4 Rank turnover . . . 10
S3.5 Rank change . . . 11
S3.6 Rank inertia . . . 12

S4 Model of rank dynamics **13**

S4.1 Model definition . . . 13
S4.2 Displacement probability . . . 14
S4.3 Approximation for displacement probability . . . 17
S4.3.1 Continuum limit for diffusion peak . . . 18
S4.4 Approximation for rank flux . . . 20
S4.5 Approximation for rank turnover . . . 23
S4.6 Approximation for rank change . . . 25
S4.7 Approximation for rank inertia . . . 26

S5 Fitting data with model **27**

S5.1 Dynamical regimes of open ranking lists . . . 30
S5.2 Fluctuations, estimation bias, and goodness of fit . . . 31
S5.3 Effect of subsampling . . . 35

S6 On the heterogeneity of rank dynamics **36**

S1 Summary of notation

We consider a *ranking list* at times $t = 0, \dots, T-1$, that is, an ordered set of N_0 elements (with N_0 constant in time) where each element i in the list at time t has a *score* $s_i(t)$, and elements are ordered across the list with decreasing score. Thus, elements and their order may change throughout time. The most important element (with the largest score) at time t has *rank* $R_t = 1$; the least important element (with the lowest score) has rank $R_t = N_0$, and the ranking is the particular order of elements and scores across ranks $R = 1, \dots, N_0$, which changes in time as elements vary their scores. We consider the case where we do not have access to the scores at all times, but only in a discrete set of T observations, separated in average by a real time interval ℓ (measured in days) [1]. We also consider the case where we may not have empirical data on the scores of all elements at all times, either because elements enter/leave the ranking at some point in time, or because score data is unavailable. Thus, there are $N_t \geq N_0$ distinct elements that have ever been in the ranking up to (and including) time t. If $N_t = N_0$ for all t the ranking list is *closed*, since elements do not leave or enter the ranking list, and if $N_t > N_0$ the ranking list is *open*. In what follows we characterize the temporal variability of ranking lists across many systems by analysing the flow of elements in and out of the ranking list as time goes by.

S2 Data description

We analyse 30 datasets in a wide range of structural and temporal scales, comprising several definitions of elements and scores. Table S1 lists the observed system size N_{T-1} (number of distinct elements ever seen in the ranking), ranking list size N_0, number of observations T, and real time interval ℓ for all datasets considered. Table S1 also includes a system classification based on the nature of the elements in the system, and the corresponding definitions of elements and scores for each system. Social systems reflect human interactions at the individual and organizational levels. Language datasets show how word usage has changed across centuries. Economic rankings illustrate value at different scales. Systems in the infrastructure category are specific to public transport and city populations. Nature datasets gather information from biological and geological phenomena. Finally, rankings in sports capture the relative performance of players and teams according to sets of rules. We now describe the datasets in more detail.

In their original state some datasets are not homogeneous in time and size, since they do not have the same real time interval between any times t and $t+1$ or the same number of elements per observation (i.e. they have a variable ranking list size). To consistently analyse all datasets, we crop the data to obtain roughly homogeneous time intervals and have a constant ranking list size across observations, while trying to retain as large T and N_0 as possible.

S2.1 Society

GitHub repositories. GitHub [1] is perhaps the most popular web-based version control repository, mostly used for source code. This dataset contains daily rankings of repositories, based on the number of users watching each project, from April 1, 2012 to December 30, 2014.

[1] The time unit is arbitrary and may be anything other than days.

Dataset	Element	Score	Measure			
			N_{T-1}	N_0	T	ℓ (days)
**Society**						
GitHub repositories [1]	repository	# watchers	450655	4773	727	1.00
The Guardian readers (recc) [2]	person	avg # recommends	29165	753	182	1.00
The Guardian readers (comm) [2]	person	# comments	18244	753	182	1.00
Enron emails [3]	person	# emails	4720	209	101	7.00
Scientists [4, 5]	person	# citations	2614	1041	45	365.25
Universities [6]	university	ARWU score [7]	140	100	14	365.23
**Languages**						
Russian [8–11]	word	frequency	281346	35494	210	365.24
Spanish [8–11]	word	frequency	233323	31750	260	365.24
German [8–11]	word	frequency	195455	22661	262	365.25
French [8–11]	word	frequency	182507	17645	367	365.24
Italian [8–11]	word	frequency	139645	20879	244	365.24
English [8–11]	word	frequency	124464	17750	334	365.24
**Economics**						
Companies [12]	company	revenue	1895	500	51	365.26
Countries [13–15]	country	complexity [13–15]	139	99	49	365.25
**Infrastructure**						
Cities (RU) [16]	city	population	1639	936	8	4383.00
Metro stations (London) [17]	station	# passengers	636	636	69	0.0104
Cities (GB) [18]	city	population	458	458	11	3652.50
Metro stations (Mexico)	station	# passengers	175	175	365	1.00
**Nature**						
Hyenas [19]	animal	association index	303	43	23	365.23
Regions JP (quake mag) [20, 21]	region	avg quake magnitude	264	264	176	28.00
Regions JP (quakes) [20, 21]	region	# quakes	264	264	176	28.00
**Sports**						
Chess players (male) [22]	person	Elo rating [23]	16568	13500	46	30.44
Chess players (female) [22]	person	Elo rating [23]	16539	12681	46	30.44
Poker players [24]	person	GPI score [25]	9799	1795	221	7.04
Tennis players [26]	person	ATP points [27]	4793	1600	400	7.00
Golf players [28]	person	OWGR points [29]	3632	1150	768	7.00
Football scorers [30]	person	# goals [31]	2397	400	53	7.04
NASCAR drivers (Busch) [32]	person	NASCAR points	676	76	34	365.24
NASCAR drivers (Winston Cup) [32]	person	NASCAR points	272	50	35	365.26
National football teams [33]	team	FIFA points [34]	210	200	71	30.44

Table S1. Datasets used in this study. Characteristics of the available datasets, including the observed system size N_{T-1}, ranking list size N_0, number of observations T, and real time interval between observations ℓ. The table includes a system type based on the nature of the elements in the ranking list, as well as the corresponding definitions of elements and scores for each system, and references regarding the dataset and score system.

The Guardian readers. The Guardian [2] is a British national daily newspaper, publishing online articles (news and opinion pieces) in diverse subjects. Users registered to the website can post comments to some of the articles, which readers may 'recommend' (by clicking a 'like' button akin to those of social network sites). Focusing on the period from November 1, 2011 till May 1, 2012, we crawl articles appearing in three sections of The Guardian ('politics', 'sport', and 'comment is free'), and rank users daily by the number of comments they write (denoted 'comm'), as well as by the average number of

recommends their comments receive (denoted 'recc').

Enron emails. The Federal Energy Regulatory Commission, during its investigation of the company Enron, made public about half a million emails from roughly 150 users, mostly senior managers. A cleaned, current version of the dataset is available online [3]. From this dataset we rank users (email accounts) by the number of emails sent, on a weekly basis.

Scientists. We use yearly citation data from journals of the American Physical Society to rank the most cited scientists by number of citations [4, 5].

Universities. ShangaiRanking Consultancy is an independent organization dedicated to higher education research. During the period 2003–2016 [6], the organization ranked top universities according to the Academic Ranking of World Universities (ARWU) score, which considers several criteria such as number of students, publications, Nobel Prizes, Fields Medals, etc. [7].

S2.2 Languages

We use a subset of the publicly available Google Books Ngram dataset [8,9], the result of the digitalization and conversion of millions of books in several languages (about 4% of all published books until 2009). From this data we extract the frequency of words by year for Russian, Spanish, German, French, Italian, and English. The original dataset is case sensitive, so we merge words with different cases. We have already analyzed the temporal variability of ranks at several scales for these languages in previous studies [10, 11].

S2.3 Economics

Companies. Since 1955, *Fortune* magazine has compiled a yearly dataset of the top 500 corporations in the world based on yearly revenue. Here we use the freely accessible archives of 1955–2005 [12].

Countries. MIT's Observatory of Economic Complexity [13] has compiled a yearly ranking dataset of the economic complexity of countries between 1962 and 2015 [14, 15], where a complexity score reflects the diversity of exports of countries around the globe.

S2.4 Infrastructure

Cities. To obtain ranking datasets of cities by population, we use data from previous studies of Russia (RU) [16] and Great Britain (GB) [18]. In Russia, urban population has decreased, while in most other countries it has increased. For Great Britain, population data corresponds to 63 primary urban areas from England, Scotland, and Wales between 1901 and 2011.

Metro stations. The London Underground has 270 stations, serving about 5 million passengers per day. For London, the ranking dataset corresponds to aggregated Oyster smart-card data of 636 station

entrances in a week of 2012, considering 15-minute intervals [17]. For Mexico City (Mexico), we requested and obtained data directly from *Sistema de Transporte Colectivo Metro*, the part of Mexican government directing metro services, and used daily entrance data for 175 stations during 2014.

S2.5 Nature

Hyenas. A 23-year field study has monitored the social relationships of a spotted hyena population in Kenya [19]. Using this dataset, we rank individual hyenas according to the sum of an association index describing the strength of the relationships with the rest of the hyenas over a particular year.

Regions JP. The Japan University Network Earthquake Catalog openly records earthquake events happening in Japan (JP) [20]. Following a previous study [21], we only consider earthquakes with Richter magnitude larger than 2.0, happening between July 1, 1985 and December 31, 1998, taking main-shocks and after-shocks as separate events. We rank administrative regions in Japan by both the monthly number of earthquakes happening in a region (denoted 'quakes'), and the average earthquake magnitude in a month (denoted 'quake mag').

S2.6 Sports

Chess players. Every month, the Fédération Internationale des Échecs (FIDE, International Chess Federation) ranks top male and female players [22] based on the Elo rating system [23].

Poker players. Weekly rankings of Poker players are listed online [24]. Rankings are based on the Global Poker Index (GPI) score [25], which takes into account players who participated in tournaments in the last 18 months, including an aging factor and the scores obtained in different tournaments.

Tennis players. The Association of Tennis Professionals (ATP) [26] has an intricate ranking system based on ATP points [27], which is updated every week to rank male players in Singles.

Golf players. The Official World Golf Ranking (OWGR) publishes weekly lists of player rankings, accumulating points achieved in tournaments in the previous two years [28]. To calculate OWGR points, the points achieved by each player on all tournaments during these two years are averaged over the number of tournaments involved [29].

Football scorers. The Football World Rankings publishes a weekly list of top scorers [30], considering the number of goals scored by each player in the previous year [31].

NASCAR drivers. We use an open dataset containing the ranking of drivers of the National Association for Stock Car Auto Racing (NASCAR), both for the Busch Series and the Winston Cup [32].

National football teams. The Fédération Internationale de Football Association (FIFA, International Federation of Association Football) [33] publishes a monthly list of national teams ranked according to

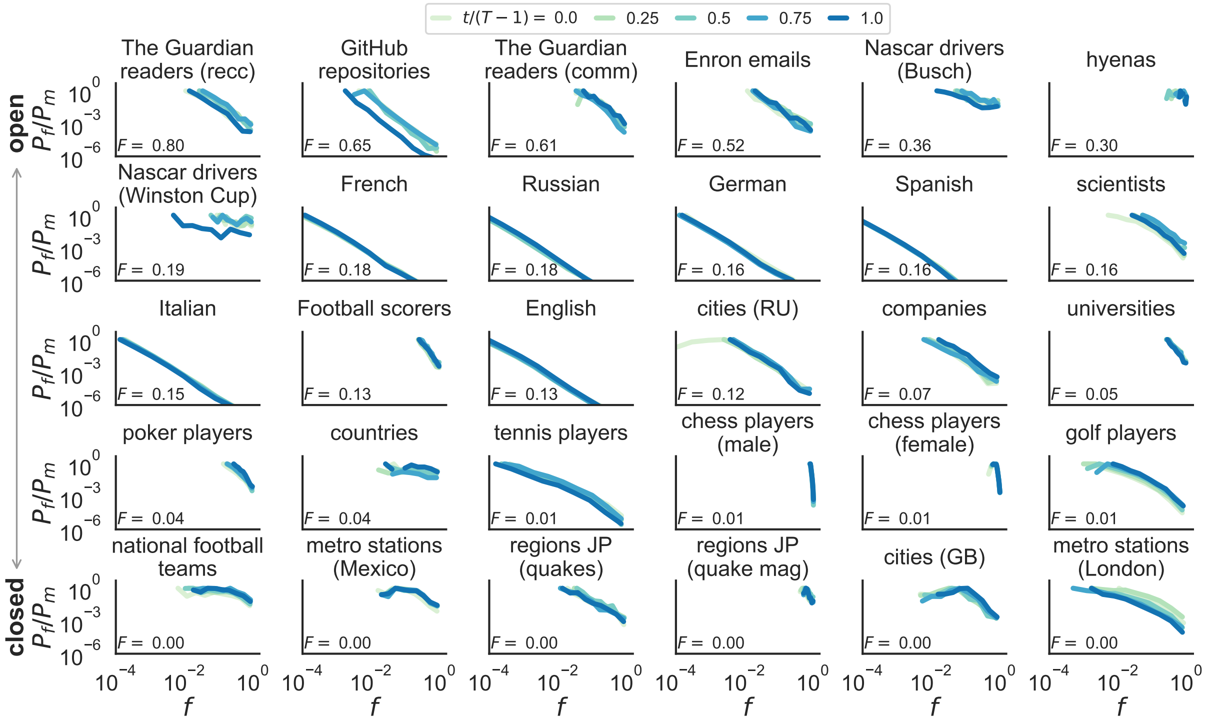

Figure S1. Fitness in open to closed ranking lists. Probability P_f that a randomly selected element i has fitness f_i at time t, normalized by the maximum value of P_f at the time (denoted P_m). We plot P_f for various times t (colored lines). To compare across observations $t = 0, \dots, T-1$, we calculate fitness as $f_i(t) = s_i(t)/s_m(t)$, with $s_i(t)$ the score of element i and $s_m(t)$ the maximum score in the ranking at time t [Eq. (S1)]. Datasets are ordered from most open (upper row) to closed (lower row) according to mean rank flux F [Eq. (S2)]. Fitness distributions are either broad (spanning orders of magnitude) or constrained to a small interval, and show wild variations across datasets.

the points obtained in the previous four years, using several criteria to determine FIFA points [34]. Data for female teams has a lower temporal scale, so we restrict our analysis to male teams.

S2.7 Acknowledgments and data availability

We acknowledge José A. Morales and Sergio Sánchez for data handling in the initial stages of the project. We are grateful for data provision to Syed Haque (GitHub repositories and Enron emails), Raj Kumar Pan and author Gerardo Iñiguez (The Guardian), Roberta Sinatra (Scientists), Roberto Murcio (Cities [RU and GB], Metro stations [London]), Gustavo Carreón (Metro stations [Mexico]), Amiyaal Ilany and Kay Holekamp (Hyenas), and Márton Karsai (Regions JP). All data is either openly accessible, can be crawled directly from the web, or can be requested directly from the previous researchers under reasonable conditions.

S3 Rank measures

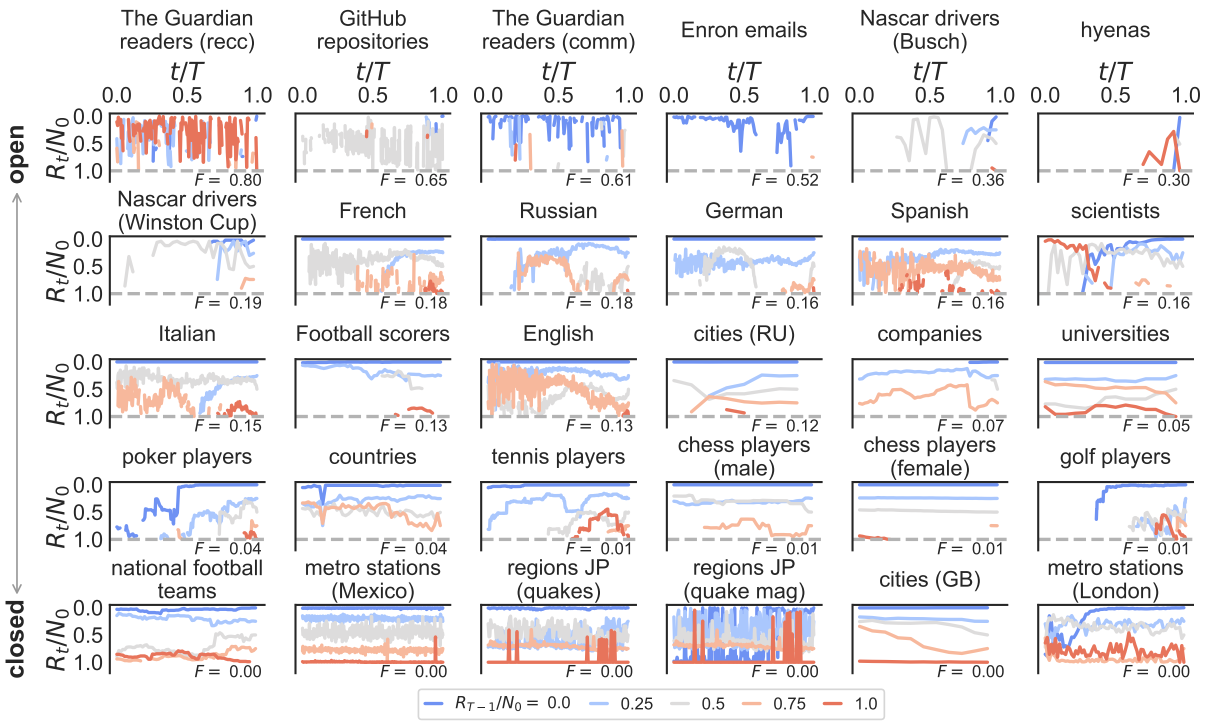

Figure S2. Rank dynamics in open to closed ranking lists. Normalized rank R_t/N_0 for selected elements in ranking as a function of normalized time t/T. Rank time series are colored according to the last observed rank of the element, R_{T-1}/N_0. The lower part of plots ($R > N_0$, below dashed lines) represents the unobserved part of an open system, containing elements without known score/rank at the time. Datasets are ordered from most open (upper row) to closed (lower row) according to mean rank flux F [Eq. (S2)]. Relatively open ranking lists are more stable at the top (low R_t) than at the bottom (high R_t). Closed ranking lists are more stable both at the top (low R_t) and bottom (high R_t), and less stable in the middle.

S3.1 Fitness

Scores $s_i(t)$ vary across elements and observations of the ranking list. To compare their distribution between times t, we normalize the score of element i as the *fitness*

$$f_i(t) = \frac{s_i(t)}{s_m(t)}, \tag{S1}$$

with $s_m(t)$ the maximum score in the ranking at time t. We define P_f as the probability that a randomly selected element i has fitness f_i at time t, and P_m as the maximum value of P_f at time t. The normalized fitness distribution P_f/P_m for each time $t = 0, \ldots, T-1$ varies greatly between systems, having either a broad or narrow functional form (Fig. S1). In most ranking lists, P_f is relatively constant in time.

S3.2 Rank dynamics

We consider the dynamics of rank $R_t = 1, \ldots, N_0$ of a given element as a function of time t. In most open ranking lists ($N_t > N_0$), the time series R_t at the top of the ranking ($R_t \ll N_0$) tend to be stable, while R_t fluctuates more as we go down in ranking (middle rows in Fig. S2). If at some time t an element is not in the ranking, its rank R_t is not observable from empirical data (because we do not know the corresponding score). For some open ranking lists even the top of the ranking is not stable, since the time

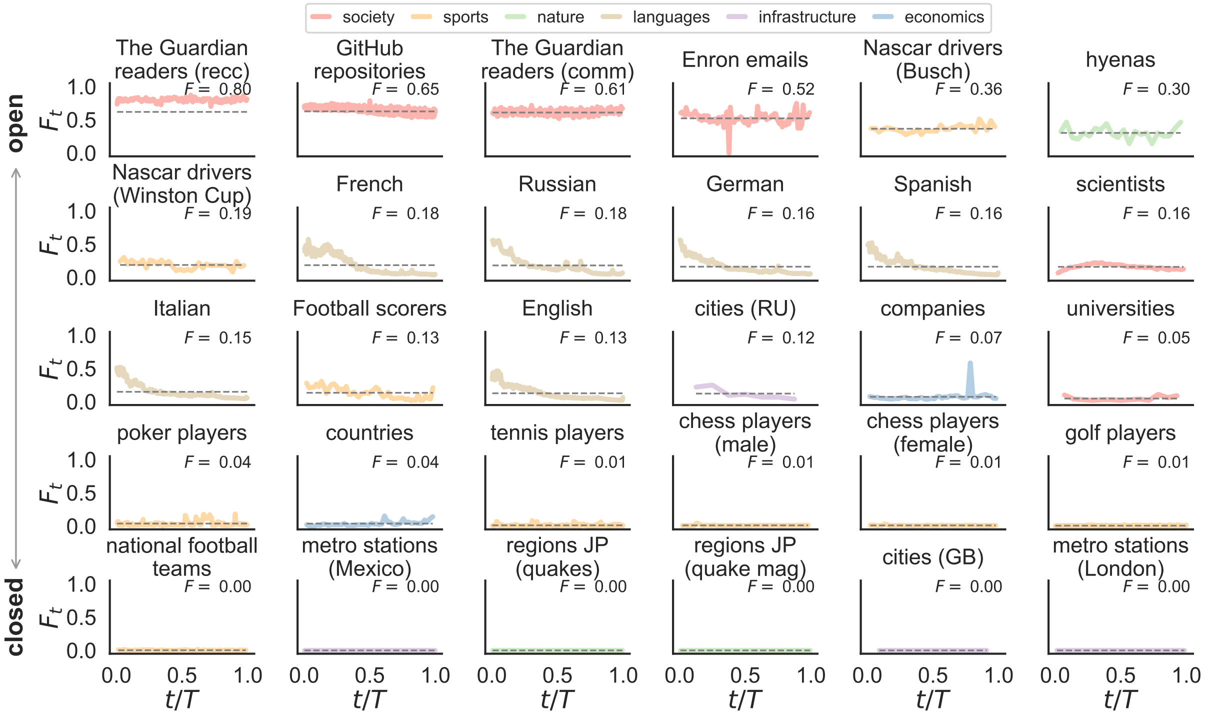

Figure S3. Rank flux in open to closed ranking lists. Flux F_t as a function of normalized time t/T, calculated as the probability that an element in (out of) the ranking list at time $t-1$ leaves (enters) the ranking at time t, averaged over all elements in the ranking at the time. Flux is shown for data (continuous lines) and the model of Section S4 with Eq. (S27) for $s = N$ (dashed lines) (for parameter fitting see Table S2 and Section S5). Datasets are ordered from most open (upper row) to closed (lower row) according to mean rank flux F [Eq. (S2)]. Flux is roughly constant over time, with occasional large deviations from the mean. In languages, flux has a decreasing trend over time. Closed ranking lists have zero flux, consistent with $N_t = N_0$.

scale T is larger than the typical lifetime of an element in the system (see, e.g., upper rows in Fig. S2, or datasets where people or companies have not been active during the whole observation period). In closed ranking lists ($N_t = N_0$), both the top and the bottom of the ranking are stable, while the middle part of the ranking shows more variation in R_t (lower rows in Fig. S2).

S3.3 Rank flux

In open ranking lists, elements flow into and out of the ranking (due to scores not being measured all the time), meaning that an element may not have a well-defined rank $R_t = 1, \dots, N_0$ for all times $t = 0, \dots, T-1$. We define *rank flux* F_t as the probability that an element in (out of) the ranking list at time $t-1$ leaves (enters) the ranking at time t, averaged over all elements in the ranking list [2]. For most open ranking lists, flux is roughly constant throughout time (upper rows in Fig. S3), i.e. F_t does not considerably deviate from its mean over time,

$$F = \frac{1}{T-1} \sum_{t=1}^{T-1} F_t. \tag{S2}$$

[2]Since the ranking size N_0 is constant in time, the number of elements that enter and leave the ranking between two consecutive observations are equal.

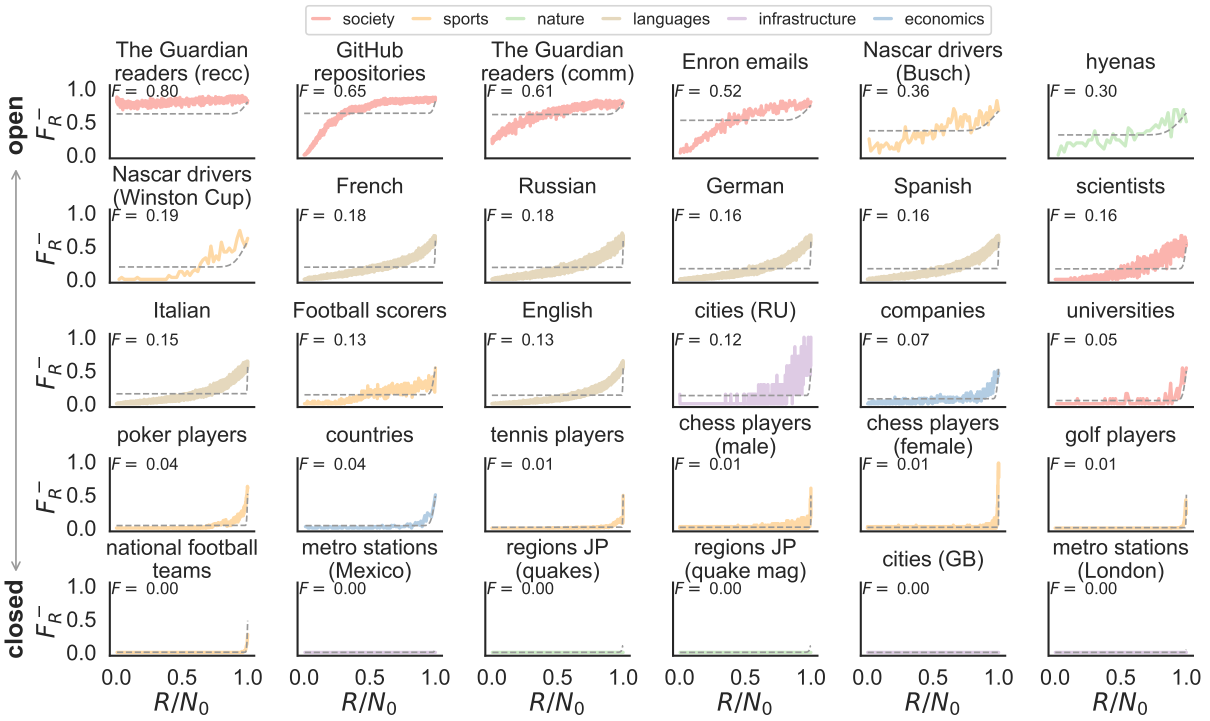

Figure S4. Rank out-flux in open systems. Out-flux F_R^- as a function of normalized rank R/N_0, defined as the probability that an element in rank R at time $t-1$ leaves the ranking at time t, averaged over time. Out-flux is shown for data (continuous lines) and the model of Section S4 with Eq. (S29) for $s = N$ (dashed lines) (for parameter fitting see Table S2 and Section S5). Datasets are ordered from most open (upper row) to closed (lower row) according to mean rank flux F [Eq. (S2)]. Out-flux has very similar qualitative behavior to in-flux in all considered datasets.

The constant trend in rank flux is sometimes disrupted by large deviations when a larger or smaller number of elements enter/leave the ranking list. In language datasets, F_t has a decreasing trend over time (Fig. S3), arguably due to the exceptionally long observation period (centuries instead of days/years, see Table S1), or to a very heterogeneous sampling of words throughout observations. Closed ranking lists have zero flux, since $N_t = N_0$ for all times t. Values of the constant flux F in all datasets are reproduced by the model of Section S4 [Eq. (S27)], except for the most open ranking list explored [The Guardian readers (recc)], where the fitting process is less precise (see Section S5).

We define *out-flux* F_R^- as the probability that an element in rank R at time $t-1$ leaves the ranking list at time t, averaged over all times $t = 1, \ldots, T-1$ (Fig. S4). Similarly, we define *in-flux* F_R^+ as the probability that an element out of the ranking list at time $t-1$ gets rank R at time t, averaged over time. Out-/in-fluxes are time averages of flux that determine what part of the ranking contributes more to the flow of elements out of (and into) it. Even though out-/in-fluxes do not need to be equal in general, they are very similar in all considered datasets. In most open systems the top of the ranking (low R) is more stable than the bottom (high R), since high out-flux tends to appear only at the bottom. This functional form is recovered by the model of Section S4 [Eq. (S29)], except for very open systems [upper rows in Fig. S4]. On the other hand, the change in concavity of out-flux (as a function of R) for some of the most open systems is not captured by the model.

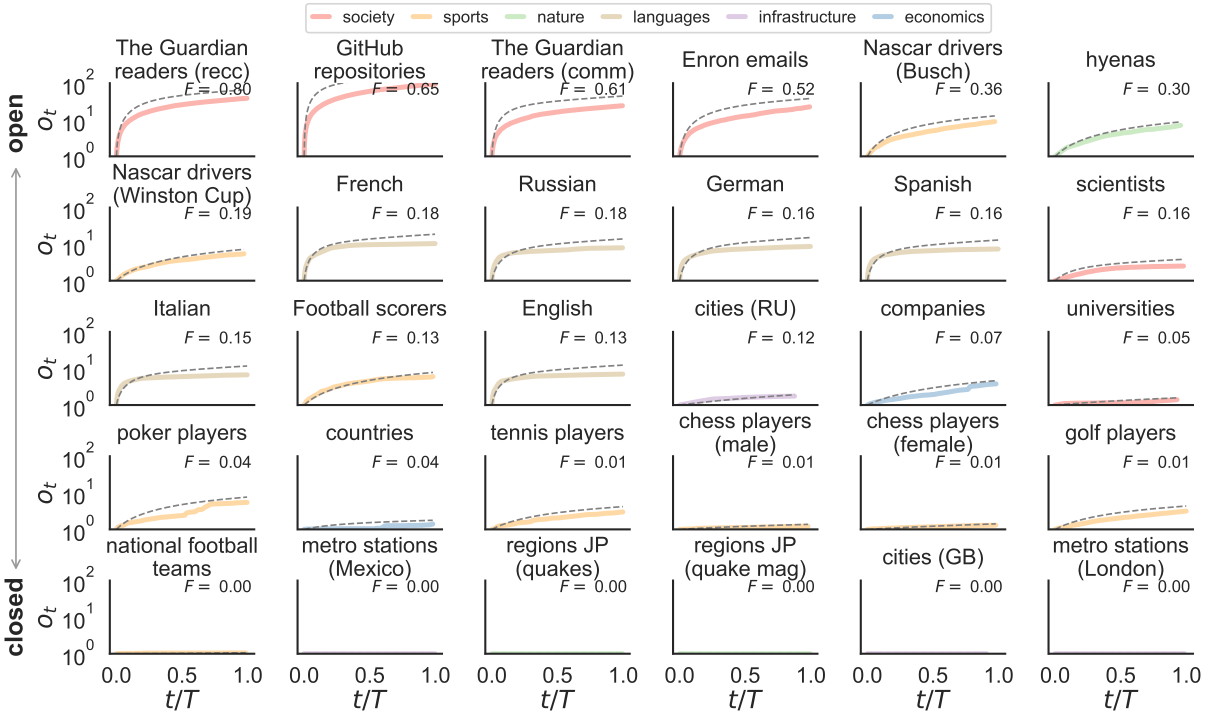

Figure S5. Rank turnover in open to closed ranking lists. Rank turnover $o_t = N_t/N_0$ as a function of normalized time t/T, defined as the number N_t of distinct elements that have been in the ranking at any time up to t, in units of ranking list size N_0 [Eq. (S3)]. Turnover is shown for data (continuous lines) and the model of Section S4 with Eq. (S38) for $s = N$ (dashed lines) (for parameter fitting see Table S2 and Section S5). Datasets are ordered from most open (upper row) to closed (lower row) according to mean rank flux F [Eq. (S2)]. Most datasets have a concave (or roughly linear) turnover in time, while closed ranking lists have constant turnover $o_t = 1$ for all t.

S3.4 Rank turnover

As mentioned in Section S1, N_t is the number of distinct elements that have ever been in the ranking list up to time t (i.e. at any $t' = 0, \dots, t$). Closed systems have $N_t = N_0$ for any t, and open systems have $N_t > N_0$ for some $t > 0$. The ranking list size N_0 is the initial condition of the time series N_t, since in our initial observation we can only measure N_0 scores. Since N_t counts the elements that have visited the ranking up until time t, it is a monotonically increasing function ($N_{t'} \leq N_t \leq N_{T-1}$ for $t' \leq t$). The value N_{T-1} is an observable proxy for the (unknown) size of the system that may increase with larger T, since more observations potentially mean access to scores of new elements (see related discussion in the model definition of Section S4.1). To compare ranking lists of different size N_0, we define the *rank turnover* of a ranking list at time t as the number of elements seen up until that time in units of ranking list size,

$$o_t = \frac{N_t}{N_0}. \tag{S3}$$

In open ranking lists, the time series o_t increases from $o_0 = 1$ to $o_{T-1} = N_{T-1}/N_0$ (see Table S1), with average slope $\dot{o}_t = (o_t - o_0)/t$ (Fig. S5). The mean turnover rate after T observations, $\dot{o} \equiv \dot{o}_{T-1} \in [0, 1]$, is a single number that characterises the turnover of a ranking list [just like the mean flux F in Eq. (S2)], from $\dot{o} = 0$ for closed systems, to $\dot{o} = 1$ for the most open system possible (where any element in the ranking list is only seen once across observations). In most open datasets considered, turnover o_t has a concave (or roughly linear) functional form in t. Our minimal model of rank dynamics introduced in

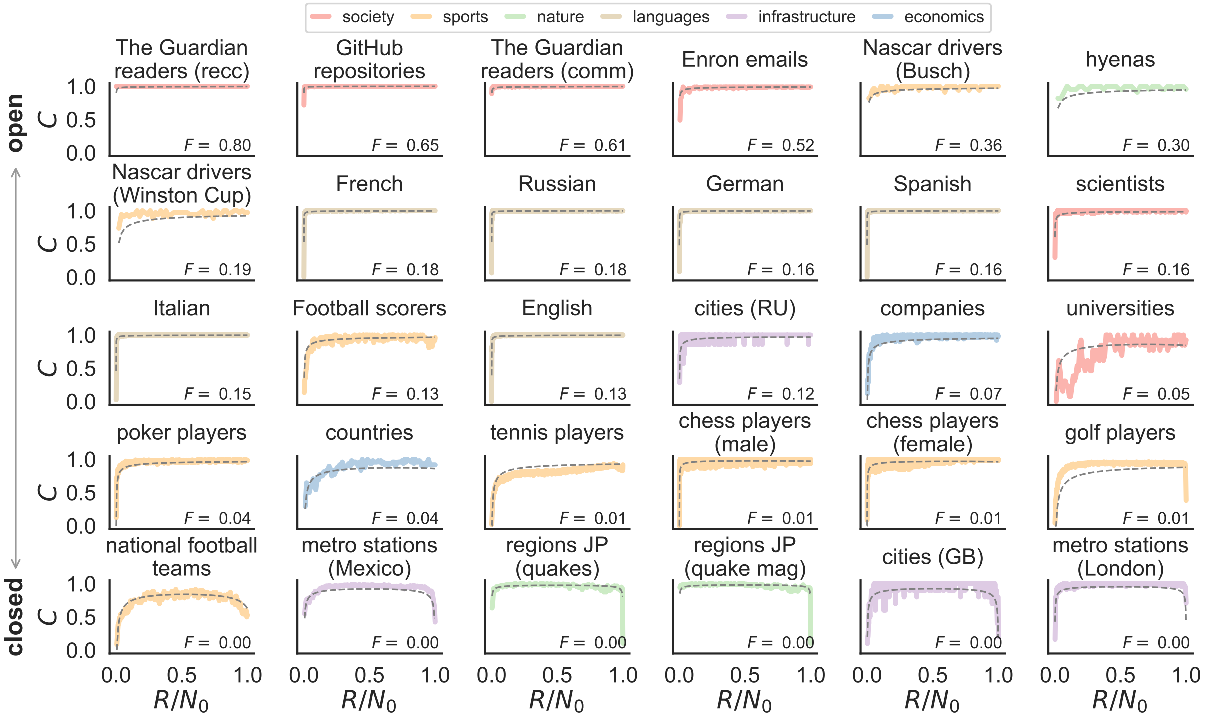

Figure S6. Rank change in open to closed ranking lists. Rank change C as a function of normalized rank R/N_0, calculated as the probability that elements in rank R at times $t-1$ and t are not the same, averaged over time. Rank change is shown for data (continuous lines) and the model of Section S4 with Eq. (S41) for $s = N$ (dashed lines) (for parameter fitting see Table S2 and Section S5). Datasets are ordered from most open (upper row) to closed (lower row) according to mean rank flux F [Eq. (S2)]. In relatively open ranking lists, rank change is asymmetric: C is lower at the top of the ranking (low R) than at the bottom (high R). In progressively more closed ranking lists, rank change turns roughly symmetric in R, with low C also at the bottom of the ranking.

Section S4 captures the qualitative behavior of turnover and its derivative [see Section S4.5, particularly Eqs. (S38)-(S40)].

S3.5 Rank change

In order to measure the stability of rank dynamics, we introduce *rank change* C as the probability that elements in rank R at times $t-1$ and t are not the same, averaged over all times $t = 1, \dots, T-1$ (Fig. S6). In most open ranking lists, rank change is an asymmetric function of R, with low values at the top of the ranking (low R) and high values elsewhere. Closed ranking lists, in turn, have symmetric C, with low rank change also at the bottom of the ranking (high R). In other words, most systems (except the most open datasets studied) have a stable ranking at the top where elements keep their rank most of the time. Yet closed ranking lists are also stable at the bottom, while open ranking lists have an unstable ranking at the bottom due to a constant flow of elements in and out of the system (Figs. S3-S4).

The qualitative behavior of C is similar to that of *rank diversity* d_R, defined as the number of distinct elements occupying rank R over all times $t = 1, \dots, T-1$, normalized by T. Rank diversity has been previously introduced and studied by us in detail, particularly in the case of ranking lists in language and sports [10, 11, 35, 36]. While both rank change and diversity capture the qualitative difference in ranking behavior between open and closed systems, rank change is more analytically tractable in our model (see Section S4). Following Eq. (S41), the model captures the functional shape and symmetry of

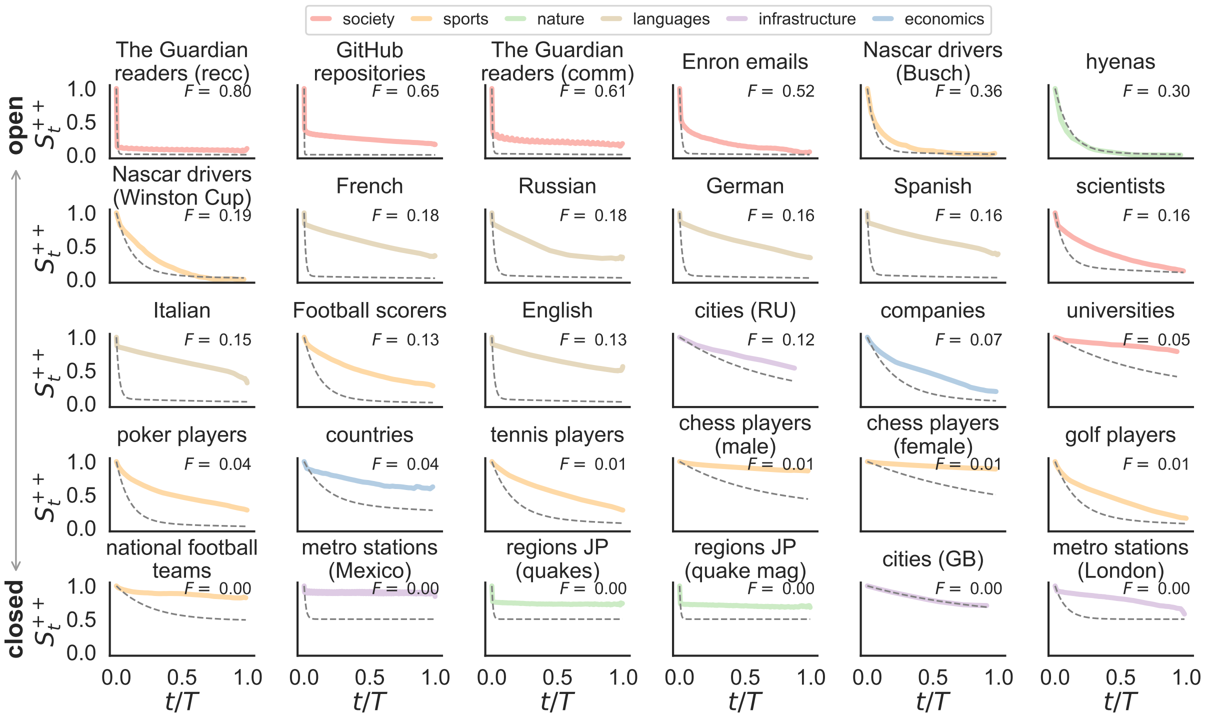

Figure S7. Inertia in open to closed ranking lists. Inertia S_t^{++} as a function of normalized time t/T, calculated as the probability that an element in the top of the ranking at time t' will stay there after t observations, averaged over t'. The top of the ranking is defined as all ranks lower than an arbitrary threshold ($R = 1, \ldots, cN_0$), set here to $c = 1/2$. Inertia is shown for data (continuous lines) and the model of Section S4 with Eq. (S43) for $s = N$ (dashed lines) (for parameter fitting see Table S2 and Section S5). Datasets are ordered from most open (upper row) to closed (lower row) according to mean rank flux F [Eq. (S2)]. In all ranking lists, the probability of staying in the top longer times decreases with t.

C for both open and closed ranking lists.

S3.6 Rank inertia

We further analyze the flux of elements within the ranking list by dividing it into two regions via an arbitrary threshold $c \in (0, 1)$: we define the *top* (+) of the ranking as all ranks lower than a threshold ($R \leq cN_0$), and the *bottom* (−) as the rest of the elements in the ranking. We define the matrix element S_t^{ij} as the probability that an element in region i will move to region j after t observations (averaged over all compatible times $t' = 0, \ldots, T - t - 1$), with $i, j \in \{+, -\}$. The matrix element S_t^{ij} characterizes the flux of elements within the ranking list over time periods of size t. We focus on *rank inertia* S_t^{++}, the probability that an element stays in the top of the ranking list over a time t. Inertia S_t^{++} is a decreasing function of t for all datasets: while in some ranking lists the probability of staying in the top is roughly constant, for most systems it decays linearly, exponentially, or with a long tail (Fig. S7). In all cases, rank inertia show the same qualitative behavior for intermediate values of the arbitrary threshold c (defining the relative sizes of the top and bottom of the ranking), although for some ranking lists, c has an effect on the functional form of S_t^{ij}. The model of Section S4 captures the decay of S_t^{++} with lag [Eq. (S43)], although there are deviations from the empirical values for most datasets.

S4 Model of rank dynamics

Here we introduce and explore in detail a minimal model for the dynamics of ranking lists. We derive approximate master equations and their solutions for both the probability of an element changing rank and some of the rank measures introduced in Section S3. Our aim is to show how closely numerical simulations of the model (and their analytical approximations) emulate microscopic and macroscopic properties of the ranking dynamics of the datasets described in Section S2.

S4.1 Model definition

Our model is simple and corresponds to the null hypothesis that rank dynamics is driven by two processes: i) *random displacements of elements* across the ranking list, leading to Lévy flight- and diffusion-like movement in rank (tuned by a model parameter $\tau \in [0, 1]$); and ii) *random replacement of elements* by new ones, leading to an increase in rank flux and turnover (tuned by a model parameter $\nu \in [0, 1]$). We assume that the model system is a list of $N \geq N_0$ elements (with N a parameter of the model), and define the normalized rank $r = R\Delta r = \Delta r, 2\Delta r, \dots, p, \dots, 1$, with $\Delta r = 1/N$ and $p = N_0/N$. If $N > N_0$ the system is larger than its ranking list, in which case we choose to 'ignore' the elements in ranks $R = N_0 + 1, \dots, N$ (despite their continuing dynamics), emulating lack of access to their scores in empirical data. Picturing rank space as a discrete line, negative/positive displacement means movement to the left/right of a rank in the line (i.e. to more/less important ranks in the ranking list; see Fig. S8) [3]. To compare time scales between model and data, we assume that the real time interval between observations at times t and $t + 1$ corresponds to N time steps in the model, defining the time step variable $s = 0, 1, \dots, N, \dots, N(T - 1)$, and thus $t = s\Delta r = 0, \Delta r, \dots, 1, \dots, T - 1$. In other words, t is a macroscopic time variable that describes the (finite number of) observations of the ranking list in the data, while s is a microscopic time variable that describes the dynamics in the model in the time between empirical observations.

At each time step s we perform two independent rank updates. The first update, happening with probability τ, will cause Lévy flight- and diffusion-like dynamics. The second update, happening with probability ν, will lead to the replacement of elements in the ranking.

For the first update, we select an element uniformly at random, remove it from the list and place it in one of the N spaces to the right or left of the remaining $N - 1$ elements. More precisely, we choose rank $R_{\text{old}} = 1, \dots, N$ uniformly at random and take its element temporarily out of the system (thus making the rank change $R \mapsto R - 1$ for elements previously in ranks $R = R_{\text{old}} + 1, \dots, N$). Then we re-introduce this element to the rank $R_{\text{new}} = 1, \dots, N$ chosen uniformly at random (making the rank change $R \mapsto R + 1$ for elements previously in ranks $R = R_{\text{new}}, \dots, N - 1$). In this way, elements initially between (and including) ranks R_{old} and R_{new} will change rank (if $R_{\text{old}} \neq R_{\text{new}}$), while elements outside this range will keep their rank [4] (Fig. S8).

In the second update, we choose a rank $R_{\text{rep}} = 1, \dots, N$ uniformly at random and replace its element with a new element from outside the system, leaving the rest of the ranks untouched. The old element is removed from the system and ceases to have any dynamics, while the new element participates in the rank updates of time step $s + 1$ and beyond (Fig. S8).

[3]Note that the discrete rank space $r = \Delta r, \dots, 1$ becomes the continuous interval $[0, 1]$ in the limit $N \to \infty$.
[4]For a mathematical treatment of similar shuffling models, see [37].

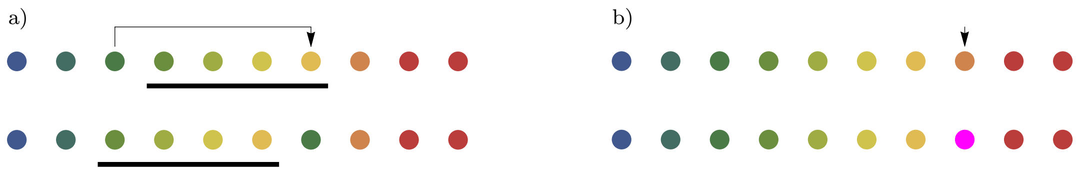

Figure S8. Model of rank dynamics. At each time step of the model dynamics we perform two independent rank updates. **(a)** In the first update (*random displacements of elements*), an element (selected uniformly at random) is reallocated to a random position, also chosen uniformly at random. This element has a (potentially long) displacement in rank space, while the elements between the previous and new position of the selected element (here underlined) move only one rank. Other elements (i.e. in the boundaries of rank space) do not change rank. **(b)** In the second update (*random replacement of elements*), we replace an element selected uniformly at random by a new element not previously in the system.

Each empirical dataset allow us to fix N_0 and T in the model (see Table S1). In what follows we will derive analytical approximations of the model dynamics for all values of the free parameters τ, ν and N (or, alternatively, τ, ν and p). Still, it's good to highlight the two parameter ranges that are sufficient to emulate the dynamics of empirical open and closed ranking lists as described in Section S3. For *closed* ranking lists we take $N = N_0$ (i.e. $p = 1$) and $\nu = 0$, so the only free parameter is τ. In this parameter range the ranking list covers the whole model system, the ranks of all elements are known at all times, no new elements enter the system, and the dynamics of rank is purely driven by Lévy flights and diffusion (as we see in Section S4.2 and Section S4.3). For *open* ranking lists we take $N = N_{T-1}$ (i.e. the model system size is the observable size of the empirical system, $p = 1/o_{T-1}$), so the free parameters are τ and ν. In this parameter range the ranking list does not cover the whole model system ($N_{T-1} > N_0$), so elements may enter/leave the ranking list from/to the unobserved ranks $R = N_0 + 1, \dots, N$ due to Lévy flights and diffusion, and new elements may replace old ones. Our model assumes that the flow of elements into and out of an open ranking list has two sources: one coming from a lack of score data outside the ranking list, and another due to birth/death of elements.

S4.2 Displacement probability

At each time step of the dynamics, with probability $\nu\Delta r$ the element in rank R is replaced by a new element, thus leaving the system. Alternatively (with probability $1 - \nu\Delta r$), the element in rank R is displaced to a new rank X in the system with probability τ, either by being randomly selected with the displacement update rule of the model (*Lévy flight*), or because of the rank change of another element (*diffusion*). Like with R, we can define the normalized rank $x = X\Delta r = \Delta r, 2\Delta r, \dots, p, \dots, 1$. We explore the temporal evolution of the model by formulating a master equation for the displacement probability $P_{x,t}$, the probability that an element in the normalized rank $r = R\Delta r$ moves to the normalized rank $x = X\Delta r$ after a time $t = s\Delta r$.

We first consider the displacement probability after a single time step of the dynamics takes place ($s = 1$), $P_x^r \equiv P_{x,\Delta r}$. An element in rank r (that is not replaced) moves to rank x with probability τ either by performing a Lévy flight (with probability L) or due to the rank change of another element (with probability D_x^r), or stays in place with probability $1 - \tau$. Then we have

$$P_x^r = (1 - \nu\Delta r)\big[\tau(L + D_x^r) + (1 - \tau)\delta_x^r\big], \tag{S4}$$

with δ_x^r a Kronecker delta. Note that for $\tau = 1$ and $\nu = 0$ (in a closed ranking list with maximum

number of displacements in a given time interval), the single-step displacement probability reduces to $P_x^r = L + D_x^r$. The Lévy probability L is straightforward to calculate: the element in rank r gets picked up by the dynamics with probability Δr, and is placed back in rank x with the same probability Δr, since both processes are uniformly random in rank space. Thus,

$$L = \begin{cases} \Delta r^2 & x \in \{\Delta r, 2\Delta r, \ldots, 1\} \\ 0 & \text{otherwise} \end{cases}. \tag{S5}$$

Eq. (S5) explains our motivation to use the term Lévy flight: a Lévy flight is a random walk where displacement length follows a heavy-tailed probability distribution; since Eq. (S5) does not depend on the normalized displacement $d = x - r$, the probability L can be thought of as a discrete power law with exponent 0 (and cut-off due to finite system size) [38, 39]. In other words, our model implements maximally heavy-tailed Lévy flights where all displacements are equally likely, beyond diffusion. If we would extend the model to allow for a non-uniform sampling of elements and ranks in the mechanisms of displacement and replacement, we expect that this exponent would become tunable.

The diffusion probability D_x^r is slightly more involved. The element in rank r moves one step to the right ($x = r + \Delta r$) if we choose an element to its right (with probability $1 - x + \Delta r$) and place it back to its left (with probability $x - \Delta r$). Conversely, the element in rank r moves one step to the left ($x = r - \Delta r$) if we choose an element to its left (with probability x) and place it back to its right (with probability $1 - x$). The element at rank r can stay in place ($x = r$) only if another element is picked and placed back to its left [with probability $(x - \Delta r)^2$] or to its right [with probability $(1 - x)^2$]. Joining these expressions we obtain

$$D_x^r = \begin{cases} x(1-x) & x = r - \Delta r \\ (x - \Delta r)^2 + (1-x)^2 & x = r \\ (x - \Delta r)(1 - x + \Delta r) & x = r + \Delta r \\ 0 & \text{otherwise} \end{cases}. \tag{S6}$$

Note that $\sum_x D_x^r = 1 - \Delta r$. In other words, the probability loss from the diffusive process in Eq. (S6) goes to the uniform contribution made by the Lévy term in Eq. (S5), meaning that the continuous limit of Eq. (S6) does not exactly correspond to a diffusion equation. Moreover, $\sum_x P_x^r = 1 - \nu \Delta r < 1$ (for $\nu > 0$), which implies a global loss of probability reflecting the fact that we replace elements in the dynamics.

Moving forward in the dynamics ($t = s\Delta r \geq 0$), we write a recurrence relation for the displacement probability $P_{x,t}$. We observe that, for an element in rank r to move to rank x after s time steps, it needs to have moved to an arbitrary rank $x' = \Delta r, \ldots, 1$ over the first $s - 1$ time steps, and then from x' to x in the last time step, i.e.,

$$P_{x,t} = \sum_{x'} P_{x',t-\Delta r} P_x^{x'} \tag{S7}$$

with a Kronecker delta as initial condition ($P_{x,0} = \delta_x^r$), since at the start of the dynamics, an element in rank r can only be in that rank ($x = r$). Note that Eq. (S7) for $s = 1$ recovers $P_{x,\Delta r} = P_x^r$ in Eq. (S4), as expected.

Let's focus for a moment on the cumulative $P_t = \sum_x P_{x,t}$, the probability that, after a time t,

the element in rank r moves to any other rank in the system. Summing up over x in Eq. (S7) gives the recurrence relation $P_t = (1 - \nu\Delta r)P_{t-\Delta r}$, with initial condition $P_0 = 1$ (due to $P_{x,0} = \delta_x^r$) and $P_{\Delta r} = 1 - \nu\Delta r$. Solving the master equation gives

$$P_t = (1 - \nu\Delta r)^s \simeq e^{-\nu s\Delta r} = e^{-\nu t}. \tag{S8}$$

The approximation in Eq. (S8) comes from using the time scale relation $t = s\Delta r$, the binomial theorem, and the power series of the exponential, and becomes exact in the limit $N \to \infty$. In other words, the probability of an element staying in the system under a dynamics of replacement ($\nu > 0$) decays exponentially with time. When there is only element displacement ($\nu = 0$), $P_t = 1$ and the ranking list is indeed closed. Consistently, Eq. (S8) is equal to the probability that an element has not been replaced at any previous time, which decays geometrically or exponentially with time (in the discrete and continuous cases, respectively).

Inserting Eqs. (S4)-(S6) into Eq. (S7) we obtain a master equation for the displacement probability after s time steps,

$$\begin{aligned} P_{x,t} = (1 - \nu\Delta r)\Big\{ P_{x,t-\Delta r} + \tau\Big[\Delta r^2 (1 - \nu\Delta r)^{s-1} + x(1-x)P_{x+\Delta r,t-\Delta r} \\ - 2\Big[x(1 - x + \Delta r) - \frac{1}{2}\Delta r^2\Big] P_{x,t-\Delta r} + (x - \Delta r)(1 - x + \Delta r)P_{x-\Delta r,t-\Delta r} \Big] \Big\}. \end{aligned} \tag{S9}$$

Eq. (S9) is reminiscent of a discretized diffusion equation in one dimension (the rank space x). The multiplicative factor $1 - \nu\Delta r$ represents the dynamics of elements being replaced, thus decreasing their probability of staying in the system as s increases. The second factor on the right hand side accounts for elements being selected by the displacement dynamics of the model and moving (possibly long) distances in rank space as Lévy flights. The last three terms on the right hand side describe a (rank-dependent) local, diffusive movement of elements in the system due to other elements performing Lévy flights. The time scale of both Lévy flights and diffusion-like movement is regulated by τ.

We can actually measure the displacement probability $P_{x,t}$ for $t = 1$ directly from empirical data as the probability that the element at rank R at time $t' - 1$ moves to rank X at time t', averaged over all times $t' = 1, \dots, T - 1$ (Fig. S9). The use of an average over time is supported by the stationarity of the empirical rank dynamics as seen in the roughly constant flux time series of most datasets (see Fig. S3). As we will see in Section S4.3 below, the empirical displacement probability resembles either a flat Lévy sea or a diffusion peak, respectively corresponding to late and early times of the rank dynamics in the model. By calculating $P_{x,t}$ in the model for $s = N$ (i.e. between times $t = 0$ and $t = 1$), we see that the model qualitatively reproduces the empirical displacement probability for most datasets and any rank [see Eq. (S22)].

As seen in Eq. (S8), the probability $P_t = \sum_x P_{x,t}$ of an element staying in the system for $\nu > 0$ decays exponentially with time, i.e. P_t is not conserved. In order to approximately solve Eq. (S9) with a diffusion ansatz (see Section S4.3), we can renormalize $P_{x,t}$ by introducing the probability distribution

$$Q_{x,t} = \frac{P_{x,t}}{(1 - \nu\Delta r)^s} \simeq P_{x,t} e^{\nu t}, \tag{S10}$$

which is indeed conserved, since $\sum_x Q_{x,t} = 1$ for all t. As before, the approximation holds for $t = s\Delta r$

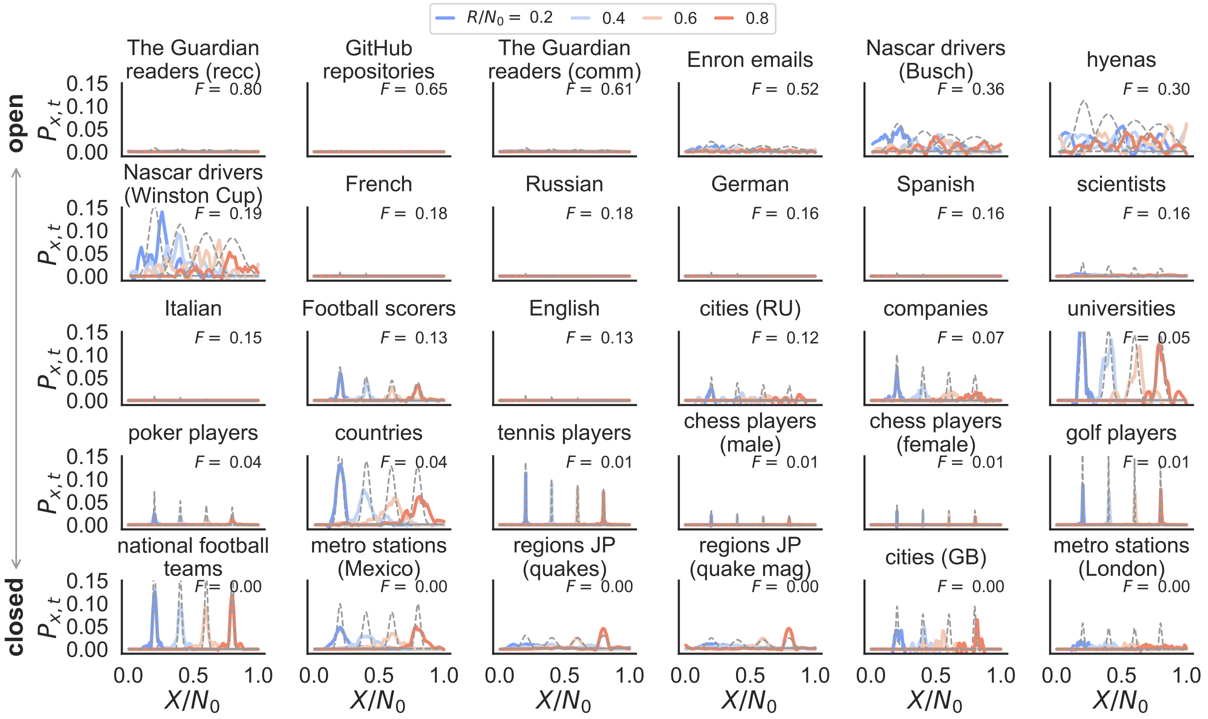

Figure S9. Displacement probability in open to closed ranking lists. Displacement probability $P_{x,t}$ for $t = 1$ as a function of normalized rank X/N_0 for various R values (colored lines), calculated as the probability that the element at rank R at time $t' - 1$ moves to rank X at time t', averaged over all times t'. $P_{x,t}$ is shown for data (continuous lines) and the model of Section S4 with Eq. (S22) for $s = N$ (dashed lines) (for parameter fitting see Table S2 and Section S5). Datasets are ordered from most open (upper row) to closed (lower row) according to mean rank flux F [Eq. (S2)]. In some datasets $P_{x,t}$ is roughly flat, reminiscent of a uniform Lévy sea, while in others the displacement probability resembles a diffusion peak (see Section S4.3). To smooth the curves, empirical data is passed through a Savitzky–Golay smoothing filter (with polynomial order 2 and filter window length ranging from $N_0/200$ to $N_0/10$).

and is exact for $N \to \infty$. Substituting Eq. (S10) into Eq. (S9) gives

$$Q_{x,t} = Q_{x,t-\Delta r} + \tau \Big\{ \Delta r^2 + x(1-x) Q_{x+\Delta r, t-\Delta r} - 2\Big[x(1-x+\Delta r) - \frac{1}{2}\Delta r^2 \Big] Q_{x,t-\Delta r} + (x-\Delta r)(1-x+\Delta r) Q_{x-\Delta r, t-\Delta r} \Big\}. \tag{S11}$$

Eq. (S11) is a simplified master equation for the displacement probability that effectively decouples the dynamics of displacement (τ) and replacement (ν) in the model. We can first look for an approximate solution $Q_{x,t}$ of the displacement dynamics governed by Eq. (S11), and then consider the replacement dynamics explicitly with $P_{x,t} = Q_{x,t} e^{-\nu t}$.

S4.3 Approximation for displacement probability

Rather than solving Eq. (S11) explicitly, we derive an approximate expression for the renormalized displacement probability $Q_{x,t}$ that becomes more accurate as $N \to \infty$. Considering the shape of Eq. (S4) (i.e. for $s = 1$), we propose an ansatz of $Q_{x,t}$ for any t separated into Lévy flight and diffusive components,

$$Q_{x,t} = L_t + D_{x,t}. \tag{S12}$$

As we will see below, the probability L_t of moving across rank space in Lévy flights is rank- and displacement-independent and grows slowly in time (a uniform *Lévy sea*), while the probability $D_{x,t}$ of diffusing in rank space due to the movement of other elements is approximately Gaussian-distributed in x (a *diffusion peak* that widens and decreases in height as time goes by) (Fig. S10).

First, we write a recurrence relation for L_t by noting that after a time $t = s\Delta r$, an element initially in rank r can only move due to diffusion within the $2s+1$ ranks around r, so $Q_{x,t} > L_t$ for $|x-r| \leq t$. Conversely, the only way for an element to move more than s ranks to each side of r is by Lévy flight, meaning $Q_{x,t} = L_t$ for $|x-r| > t$. Rewriting Eq. (S11) for $|x-r| > t$ we obtain

$$L_t = \tau\Delta r^2 + (1-\tau\Delta r)L_{t-\Delta r}, \tag{S13}$$

a recurrence relation for L_t with initial condition $L_0 = 0$ (since at the start of the dynamics no Lévy flights have yet occurred), which consistently gives $L_{\Delta r} = \tau L$ as in Eqs. (S4)-(S5). Eq. (S13) can be directly solved and gives an exact expression for the probability of moving across rank space in Lévy flights,

$$L_t = \Delta r[1-(1-\tau\Delta r)^s] \simeq \Delta r(1-e^{-\tau t}), \tag{S14}$$

with $t = s\Delta r$ and the approximation improving as $N \to \infty$. In other words, the Lévy sea in Eq. (S14) is a uniform probability (both in ranks r, x and displacement $d = x - r$) that increases asymptotically from $\tau\Delta r^2$ to Δr as $t \to \infty$.

By inserting Eqs. (S12)-(S13) into Eq. (S11), we find a master equation for the probability $D_{x,t}$ of diffusing in rank space due to the movement of other elements,

$$D_{x,t} = D_{x,t-\Delta r} + \tau\Big\{x(1-x)D_{x+\Delta r,t-\Delta r} - 2\Big[x(1-x+\Delta r) - \frac{1}{2}\Delta r^2\Big]D_{x,t-\Delta r} + (x-\Delta r)(1-x+\Delta r)D_{x-\Delta r,t-\Delta r}\Big\}, \tag{S15}$$

with initial condition $D_{x,0} = \delta_x^r$. Just like Eq. (S11), Eq. (S15) is difficult to solve exactly for any t. We can, however, write an expression for $A_t = \sum_x D_{x,t}$, the area under the diffusion peak [disregarding the Lévy sea; see Eq. (S12)]. First, we know that $\sum_x Q_{x,t} = 1$ due to its definition in Eq. (S10). Then, by plugging Eq. (S14) into this normalization condition we obtain

$$A_t = \sum_x D_{x,t} = (1-\tau\Delta r)^s \simeq e^{-\tau t}, \tag{S16}$$

with $t = s\Delta r$ and the approximation getting better as $N \to \infty$. In other words, $L_t = \Delta r(1 - A_t)$. Intuitively, the area A_t of the diffusion peak decreases exponentially as time goes by, 'leaking probability' into a Lévy sea that increases in height with t.

S4.3.1 Continuum limit for diffusion peak

We may find an explicit, approximate solution for the diffusion peak $D_{x,t}$ by analyzing the continuum limit of Eq. (S15) as $N \to \infty$. As mentioned in Section S4.1, in order to match the time scales between model (s) and data (t) we take $t = s\Delta r$ with $\Delta r = 1/N$. We also use the ansatz $Q_{x,t} = L_t + D_{x,t}$ with the Lévy sea L_t given by Eq. (S14). Since the diffusion peak leaks probability into the Lévy sea as time

goes by [see Eq. (S16)], we further propose the ansatz

$$D_{x,t} = D(x, s\Delta r)\Delta r \simeq D(x,t)dx, \tag{S17}$$

where $D(x,t)$ is an unknown probability density, x is taken as a continuous variable, and the approximation improves for larger N. Inserting Eq. (S17) into Eq. (S15) we obtain a master equation for $D(x,t)$,

$$\frac{\partial D}{\partial t} = \alpha x(1-x)\frac{\partial^2 D}{\partial x^2}. \tag{S18}$$

Eq. (S18) is a diffusion-like equation with a quadratic, rank-dependent diffusion coefficient $\alpha x(1-x)$ and $\alpha \simeq \tau\Delta r$ (for large but finite N). Note that the probability density $D(x,t)$ decreases in time as the Lévy sea increases, so Eq. (S18) is not a standard diffusion equation. If we would modify Eq. (S6) to force $A_t = 1$ by, say, adding a term Δr in D_r^r, we would obtain a diffusion equation in which the rank-dependent coefficient is inside the first rank derivative. The initial condition $D_{x,0} = \delta_x^r$ in Eq. (S15) leads to the initial condition $D(x,0) = \delta(x - x_0)$ (with $x_0 \equiv r \in [0,1]$ the initial rank of the element) and to the Dirichlet boundary conditions $D(0,t) = D(1,t) = 0$. Notice that Eq. (S18) is the Wright–Fisher equation [40], a continuous model of genetic drift that has been extensively studied from a mathematical point of view (see [41–43] and references therein).

We can solve Eq. (S18) exactly by separation of variables. Proposing the ansatz $D(x,t) = u(x)v(t)$ leads to $v(t) = v(0)e^{-\lambda t}$ with λ a separation constant, while $u(x)$ needs to satisfy the eigenvalue equation

$$\alpha x(1-x)u'' + \lambda u = 0. \tag{S19}$$

By using the Frobenius method and the boundary conditions of Eq. (S18), we determine the allowed values of λ implicitly and the associated eigenfunctions $u(x)$ as infinite series, over which the initial condition $D(x,0)$ can be expanded to obtain the particular solution of Eq. (S18) we are looking for.

However, it may be more instructive to find a closed-form approximation for $D(x,t)$ that lets us qualitatively understand the behaviour of the displacement probability in rank and time. In a small enough interval around the initial rank $x_0 = r$, we can approximate $x(1-x) \simeq x_0(1-x_0)$ in Eq. (S18), which leads to the standard diffusion equation with diffusion coefficient $\alpha x_0(1-x_0)$. Then, the fundamental solution (in the infinite rank domain $-\infty < x < \infty$) for $D(x,0) = \delta(x - x_0)$ is $G(x_0, \sigma_t^2)$, the Gaussian distribution with mean x_0 and time-dependent standard deviation $\sigma_t = \sqrt{2\alpha x_0(1-x_0)t}$, which spreads symmetrically around x_0 as time goes by [5]. This allows us to write

$$D(x,t) \simeq e^{-\tau t}G(x_0, \sigma_t^2) = e^{-\tau t}\frac{1}{\sigma_t\sqrt{2\pi}}\exp\left[-\frac{1}{2}\left(\frac{x-x_0}{\sigma_t}\right)^2\right], \tag{S20}$$

where the term $e^{-\tau t}$ is necessary for the Gaussian approximation to comply with Eq. (S16).

Finally, we derive an approximate expression for the displacement probability $P_{x,t} = Q_{x,t}e^{-\nu t}$ that becomes more accurate as $N \to \infty$. Inserting Eq. (S20) into Eq. (S17), and using the ansatz of Eq. (S12)

[5] Note that the Gaussian approximation fails progressively as we approach the 'tails' of the solution at $x \simeq 0, 1$, both due to the failing assumption $x(1-x) \simeq x_0(1-x_0)$ and to the mismatch between the boundary conditions of Eq. (S18) and the infinite rank domain of Eq. (S20). The overall effect is more accuracy for intermediate values of $x_0 = r$ rather than at the extremes of the interval $[0,1]$ (Fig. S10).

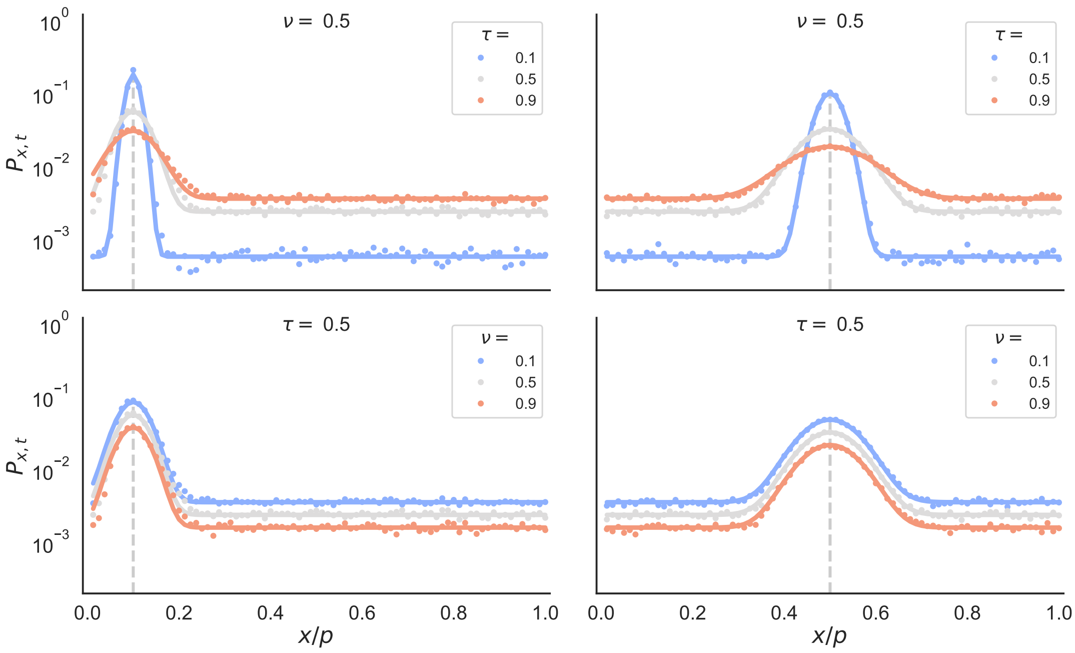

Figure S10. Displacement probability in model. Displacement probability $P_{x,t}$ as a function of normalized rank x/p for $t = 1$ ($s = N$), with $N = 10^2$ and $p = 0.8$, in numerical simulations of the model (dots) and the analytical approximation of Eq. (S22) (lines). **(top row)** Dependence of $P_{x,t}$ on τ for fixed $\nu = 0.5$, with initial rank $r = 0.08$ (left) and 0.4 (right). As τ increases, the diffusion peak around $x = r$ (dashed line) widens and decreases in height, while the uniform Lévy sea grows according to Eq. (S14). **(bottom row)** Dependence of $P_{x,t}$ on ν for fixed $\tau = 0.5$, with initial rank $r = 0.08$ (left) and 0.4 (right). As ν increases, the displacement probability is exponentially lower due to element replacement [see Eq. (S8)]. Eq. (S22) is accurate even for relatively low N. Note that the probability peak is less diffusive at the extremes of rank space (left) than at the center (right). Simulations are averages over 10^5 realizations (using $T = 10$).

alongside Eq. (S14) we obtain

$$P_{x,t} \simeq e^{-\nu t} \left[L_t + e^{-\tau t} G(r, \sigma_t^2) \Delta r \right], \tag{S21}$$

with $t = s\Delta r$, or writing everything explicitly,

$$P_{x,t} \simeq e^{-\nu t} \left[\Delta r (1 - e^{-\tau t}) + \sqrt{\frac{\Delta r}{4\pi\tau r(1-r)t}} \exp\left(-\frac{(x-r)^2}{4\tau r(1-r)t\Delta r} - \tau t \right) \right]. \tag{S22}$$

Eq. (S21) captures the approximate behavior of the displacement probability $P_{x,t}$ intuitively: a Gaussian diffusion peak $G(r, \sigma_t^2)$ widening in time and leaking probability to the uniform Lévy sea L_t, all regulated by an exponential loss in probability due to new elements. Eq. (S22) is a good approximation for $P_{x,t}$ even for relatively low $N = 10^2$, as we can see by comparing with numerical simulations of the model described in Section S4.1 for $s = N$ (i.e. $t = 1$) (Fig. S10). Eq. (S22) also shows that the model captures the displacement probability of the empirical datasets (see Fig. S9).

S4.4 Approximation for rank flux

Beyond the microscopic description of the dynamics given by the displacement probability $P_{x,t}$, we also explore the temporal evolution of the model by approximating the rank measures introduced in Section S3

with closed expressions. We start with the mean rank flux F, measured for data as the probability that any element in the ranking list at time $t-1$ leaves the ranking at time t, averaged over all recorded (observable) elements in the ranking list and over time [see Section S3.3 and Eq. (S2)]. To find F in the model, we define the time-dependent flux F_t as the probability that a given element in rank $r \leq p$ leaves any of these ranks after time $t = s\Delta r$ (either by displacement or by replacement). Following Eq. (S10), flux is given by

$$F_t = 1 - \sum_{x=\Delta r}^{p} P_{x,t} = 1 - (1-\nu\Delta r)^s \sum_{x=\Delta r}^{p} Q_{x,t}. \tag{S23}$$

where the step size in the sum is Δr, i.e. $x = \Delta r, 2\Delta r, \ldots, p$.

We now find a master equation for the partial cumulative $Q_{[a,b],t} \equiv \sum_{x=a}^{b} Q_{x,t}$ for arbitrary $a, b = \Delta r, \ldots, 1$ and $b > a$ [6]. Using Eq. (S11), summing over x, and changing dummy indices, we obtain

$$Q_{[a,b],t} = (1-\tau\Delta r) Q_{[a,b],t-\Delta r} + \tau\Big[(b-a+\Delta r)\Delta r + (a-\Delta r)(1-a+\Delta r)(Q_{a-\Delta r,t-\Delta r} - Q_{a,t-\Delta r}) + b(1-b)(Q_{b+\Delta r,t-\Delta r} - Q_{b,t-\Delta r})\Big]. \tag{S24}$$

Eq. (S24) is not closed on the cumulative $Q_{[a,b],t}$, since it depends directly on the renormalized displacement probability (last two terms on the right side of the equation). To close the master equation we make the approximations $Q_{a-\Delta r,t} \simeq Q_{a,t}$ and $Q_{b+\Delta r,t} \simeq Q_{b,t}$ for all t (accurate as long as the diffusion peak of $P_{x,t}$ is far enough from a or b), which leads to

$$Q_{[a,b],t} = (1-\tau\Delta r) Q_{[a,b],t-\Delta r} + \tau(b-a+\Delta r)\Delta r. \tag{S25}$$

We go back to the particular case of the flux F_t. With $a = \Delta r$, $b = p$, and the initial condition $Q_{[\Delta r,p],0} = 1$ (since the element starts off within the ranking list), we can solve Eq. (S25) using common formulas for geometric series to obtain

$$\sum_{x=\Delta r}^{p} Q_{x,t} = p + (1-p)(1-\tau\Delta r)^s \simeq p + (1-p)e^{-\tau t} \tag{S26}$$

for $t = s\Delta r$ and sufficiently large N. Due to the approximation in Eq. (S25), $Q_{[a,b],t}$ and F_t no longer depend explicitly on r. For $t = 1$ (i.e. $s = N$) we finally obtain

$$F \simeq 1 - e^{-\nu}[p + (1-p)e^{-\tau}]. \tag{S27}$$

Additionally, we define $F = 0$ for $p = 0$ for consistency. Eq. (S27) is an analytical approximation for the probability that any element in the ranking list at time $t-1$ leaves the ranking at time t, according to our model. Intuitively, flux F increases with τ and ν, since more displacements or replacements make it more likely that elements will leave the ranking list. Conversely, larger p makes the ranking list longer (relative to system size) and thus less likely to lose elements due to out-flux. Eq. (S27) is a good approximation of F for all parameters considered, as we can see by comparing with numerical simulations of the model described in Section S4.1 (Fig. S11). Eq. (S27) also shows that the model reproduces the empirical flux of most datasets (see Fig. S3).

[6] This general notation will be useful to determine both the flux F_t via Eq. (S23) by setting $a = \Delta r$ and $b = p$, as well as the inertia S_t^{++} via Eq. (S42) by setting $a = \Delta r$ and $b = cp$ (see Section S4.7 below).

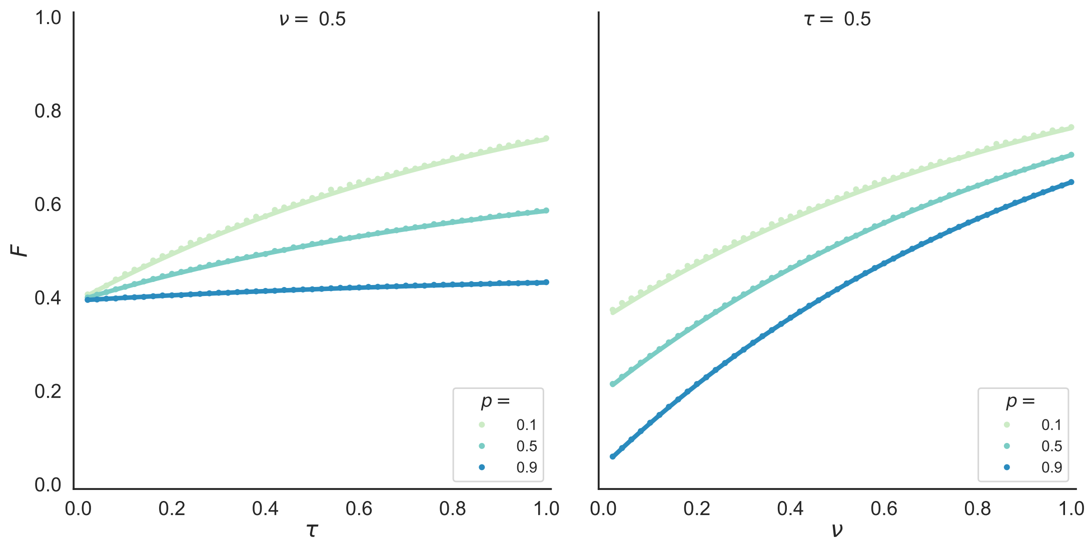

Figure S11. Rank flux in model. Rank flux F as a function of τ for fixed ν **(left)**, and as a function of ν for fixed τ **(right)**, in both numerical simulations of the model (dots) and the analytical approximation of Eq. (S27) (lines) for varying ranking list size p. Rank flux increases with both τ and ν, since the displacement/replacement dynamics takes elements out of the ranking list. A relatively longer ranking list (large p) makes it less likely that elements leave the ranking list. Simulations are averages over 10^2 realizations (with $T = 10$) in a system of size $N = 10^4$.

As described in Section S3.3, in the data we can also measure what part of a ranking list contributes most to the flow of elements out of it by calculating the out-flux F_R^-: the probability that the element in rank R at time $t-1$ leaves the ranking list at time t, averaged over all observed times. Similarly, in the model we define out-flux $F_{r,t}^-$ as the probability that the element in the normalized rank r leaves the ranking list after time $t = s\Delta r$. We can compare F_R^- in the data with $F_{r,t}^-$ for $t = 1$ (i.e. $s = N$) in the model. An element contributes to out-flux either because it is replaced with a new element, or because it moves out of the ranking list via the displacement dynamics. Thus,

$$F_{r,t}^- = 1 - (1 - \nu \Delta r)^s + \sum_{x=p+\Delta r}^{1} P_{x,t}, \tag{S28}$$

where the displacement probability $P_{x,t}$ is given explicitly by Eq. (S22) in the limit of large N. Approximating the sum in Eq. (S28) by an integral in the interval $x \in [p, \infty]$, changing variables and integrating by parts, we obtain

$$F_{r,t}^- \simeq 1 - e^{-\nu t} \left\{ 1 - (1-p)(1 - e^{-\tau t}) - \frac{1}{2} e^{-\tau t} \left[1 - \operatorname{erf}\left(\frac{p - r}{2\sqrt{\tau r (1-r) t \Delta r}} \right) \right] \right\}, \tag{S29}$$

with $t = s\Delta r$ and erf the error function. In the case $\tau = 0$, $P_x^r = (1 - \nu \Delta r)\delta_x^r$ in Eq. (S4) leads to $F_{r,t}^- \simeq 1 - e^{-\nu t}$, i.e. a constant out-flux with respect to rank r. Eq. (S29) is a very good approximation for out-flux in the model, showing how $F_{r,t}^-$ increases with both τ and ν (due to enhanced displacement and replacement dynamics), see Fig. S12. Out-flux is also relatively constant across the ranking list, except for bottom ranks ($r \sim p$) where it increases as we approach the end of the ranking list. Eq. (S29) reproduces the empirical out-flux of several datasets (Fig. S4).

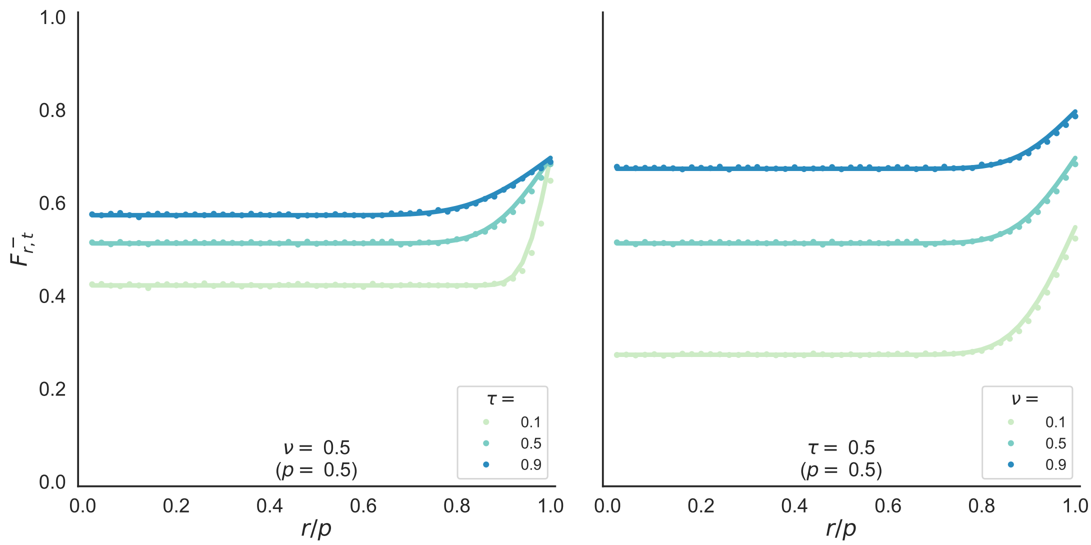

Figure S12. Rank out-flux in model. Rank out-flux $F^-_{r,t}$ as a function of normalized rank r/p for $t = 1$ ($s = N$) in both numerical simulations of the model (dots) and the analytical approximation of Eq. (S29) (lines) for fixed ranking list size p. Results correspond to varying τ for fixed ν **(left)**, and to varying ν for fixed τ **(right)**. Rank out-flux increases with both τ and ν, since the displacement/replacement dynamics takes elements out of the ranking list. $F^-_{r,t}$ is also relatively constant across the ranking list, except for bottom ranks ($r \sim p$) where out-flux increases. Simulations are averages over 10^5 realizations (with $T = 10$) in a system of size $N = 10^2$.

S4.5 Approximation for rank turnover

Here we derive an approximate, closed expression for the rank turnover $o_t = N_t/N_0$, where N_t is the number of distinct element that have been in the ranking list up to (and including) time t [see Eq. (S3)]. Similarly, in the model N_t is the number of distinct elements that have been in the ranking list ($r = \Delta r, \dots, p$) at any time step $s' \leq s$ (with $t = s\Delta r$). Thus, our task is to find an explicit expression for N_t. We start by introducing the probability p_t that a randomly chosen element has been in the ranking list at any time $t' \leq t$,

$$p_t = \frac{N_t}{M_t}, \tag{S30}$$

where M_t is the number of distinct elements that have been in the whole system at any time $t' \leq t$. Since the replacement dynamics adds one new element every time step with probability ν, on average we have $M_t \simeq N + \nu s$, which leads to

$$N_t \simeq (N + \nu s) p_t. \tag{S31}$$

In order to find p_t, we write a master equation for the probability $p_{x,t}$ that the element in any rank x of the model system [7] has been in the ranking list at any time $t' \leq t$, which we average to obtain $p_t = \Delta r \sum_x p_{x,t}$.

We first note that the initial condition for $p_{x,t}$ is

$$p_{x,0} = \begin{cases} 1 & x = \Delta r, \dots, p \\ 0 & \text{otherwise} \end{cases}, \tag{S32}$$

a step function over rank space. Within the ranking list, $p_{x,t}$ is always 1 and does not change in time,

[7] $x = \Delta r, \dots, 1$.

i.e., $p_{x,t} = p_{x,t-\Delta r} = 1$ for $x = \Delta r, \ldots, p$ and $t \geq \Delta r$. In the rest of the system, $p_{x,t}$ increases from its initial condition 0. We now write the corresponding equation for elements outside of the ranking list, in a similar way to Eq. (S7). For an element in rank x to have ever been in the ranking list, it must have belonged to the ranking list at some point in its dynamics, and finally moved from some rank r to rank x in one time step. We can thus write

$$p_{x,t} = \sum_{r=\Delta r}^{1} P_x^r p_{r,t-\Delta r}, \qquad \forall x > p. \tag{S33}$$

With Eqs. (S4)-(S6) and some simplification we obtain a master equation for $p_{x,t}$,

$$\begin{aligned} p_{x,t} = (1-\nu\Delta r)\Big\{ & p_{x,t-\Delta r} + \tau\Big[p_{t-\Delta r}\Delta r + x(1-x)p_{x+\Delta r,t-\Delta r} \\ & - 2\Big[x(1-x+\Delta r) - \frac{1}{2}\Delta r^2\Big]p_{x,t-\Delta r} + (x-\Delta r)(1-x+\Delta r)p_{x-\Delta r,t-\Delta r}\Big]\Big\}, \qquad \forall x > p. \end{aligned} \tag{S34}$$

Eq. (S34) is a discrete, diffusion-like equation similar to Eq. (S9), except that a term with $p_{t-\Delta r}$ couples the equations for all x. The temporal evolution of $p_{x,t}$ starts from a step function in rank space at $t = 0$ [Eq. (S32)], after which the front $p_{p,t} = 1$ propagates to larger ranks $x > p$, making $p_{x,t}$ increase. At the same time, the replacement dynamics (regulated by ν) decreases the probability that elements visit the ranking list. The asymptotic final state of the dynamics ($p_{x,t}$ for $t \to \infty$) depends on the parameters τ, ν, and p [see Eq. (S37) below], as well as on the rank x.

Instead of solving Eq. (S34) exactly, we find an approximate analytical solution for the corresponding master equation of p_t. Summing up over x both Eq. (S34) and the trivial solution for $x = \Delta r, \ldots, p$ [Eq. (S32)], we obtain

$$p_t = p\Big[1 - (1-\nu\Delta r)(1-\tau\Delta r)\Big] + (1-\nu\Delta r)(1-p\tau\Delta r)p_{t-\Delta r}, \tag{S35}$$

where we have made the approximation $1 = p_{p,t} \simeq p_{p+\Delta r,t}$ in order to close the equation. We explicitly solve Eq. (S35) by recursion from the initial condition p via a geometric series,

$$\frac{p_t}{p} = e^{-(\nu+p\tau)t} + \frac{\nu+\tau}{\nu+p\tau}\Big(1 - e^{-(\nu+p\tau)t}\Big), \tag{S36}$$

which leads to the asymptotic value

$$\frac{p_t}{p} = \frac{\nu+\tau}{\nu+p\tau}, \qquad t \to \infty. \tag{S37}$$

Eq. (S37) clarifies the range of accuracy of the approximation $p_{p+\Delta r,t} \simeq 1$. Depending only on the parameters of the model and as long as t is sufficiently large, if the fraction in the right hand side of Eq. (S37) is close to $1/p$, then Eq. (S35) is an accurate master equation for p_t. This typically happens for small τ or ν (see Fig. S13), or for p close to 1.

With Eq. (S31), Eq. (S3), and the explicit expression for p_t in Eq. (S36), we finally obtain

$$o_t \simeq (1+\nu t)\left[e^{-(\nu+p\tau)t} + \frac{\nu+\tau}{\nu+p\tau}\Big(1 - e^{-(\nu+p\tau)t}\Big)\right], \tag{S38}$$

with $t = s\Delta r$ and sufficiently large N, an analytical approximation for the fraction of elements in the

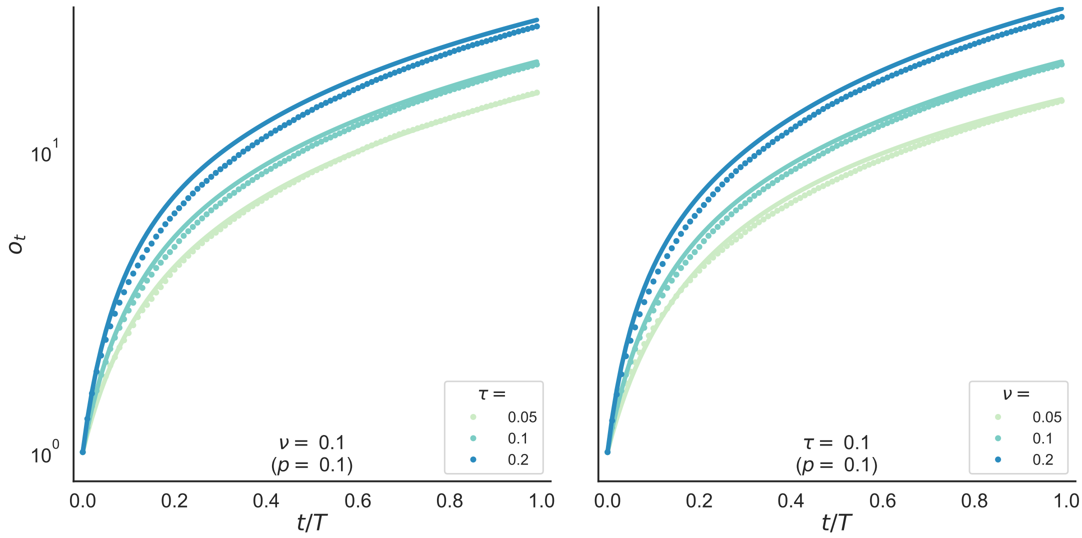

Figure S13. Rank turnover in model. Rank turnover $o_t = N_t/N_0$ as a function of normalized time t/T in both numerical simulations of the model (dots) and the analytical approximation of Eq. (S38) (lines) for fixed ranking list size p. Results correspond to varying τ for fixed ν **(left)**, and to varying ν for fixed τ **(right)**. Rank turnover has a concave shape for early times and becomes asymptotically linear. The approximation of Eq. (S38) is more accurate for small τ or ν (shown here), or for p close to 1. Simulations are averages over 10^4 realizations (with $T = 100$) in a system of size $N = 10^2$.

model that visit its ranking list as time goes by. Eq. (S38) recovers turnover in numerical simulations of the model (Fig. S13). In agreement with empirical measurements of o_t (see Fig. S5), Eq. (S38) starts off with a concave shape from its initial condition $o_0 = 1$, and has a linear behavior for long times,

$$o_t \simeq \frac{\nu + \tau}{\nu + p\tau}(1 + \nu t), \qquad t \gg 0. \tag{S39}$$

where the slope of the line is regulated by the same fraction we see in Eq. (S37). Moreover, the mean turnover rate after t observations, $\dot{o}_t = (o_t - o_0)/t$, has an asymptotic value $\dot{o} \equiv \dot{o}_{T-1}$ given by

$$\dot{o} \simeq \nu \frac{\nu + \tau}{\nu + p\tau}, \qquad T \gg 0. \tag{S40}$$

S4.6 Approximation for rank change

The approximation for the displacement probability $P_{x,t}$ in Eq. (S22) leads us directly to an explicit expression for the rank change C, defined for data as the probability that elements in rank R at times $t - 1$ and t are not the same, averaged over all times (see Section S3.5 and Fig. S6). In the model we define C_t as the probability that an element located in rank r changes place after time t (we will finally compare C with C_t for $t = 1$, i.e. $s = N$). Since $C_t = 1 - P_{r,t}$, we have

$$C_t \simeq 1 - e^{-\nu t} \left[\Delta r \left(1 - e^{-\tau t}\right) + \sqrt{\frac{\Delta r}{4\pi\tau r(1-r)t}} e^{-\tau t} \right] \tag{S41}$$

for $t = s\Delta r$ and sufficiently large N. Eq. (S41) is a good approximation for rank change in the model that fails slightly at the extremes of the ranking list (Fig. S14). In agreement with empirical data (see Fig. S6), C_t has a symmetric concave shape as a function of r, which looks asymmetric if the system

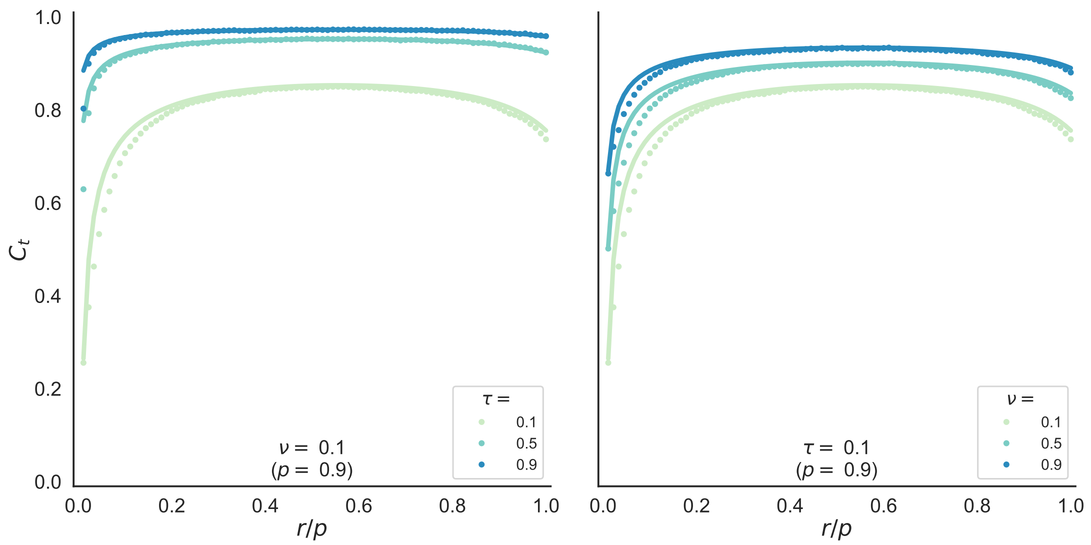

Figure S14. Rank change in model. Rank change C_t as a function of normalized rank r/p for $t = 1$ ($s = N$) in both numerical simulations of the model (dots) and the analytical approximation of Eq. (S41) (lines) for fixed ranking list size p. Results correspond to varying τ for fixed ν **(left)**, and to varying ν for fixed τ **(right)**. Rank change increases with both τ and ν, since the displacement/replacement dynamics moves elements away from their previous ranks. C_t has a symmetric concave shape across the ranking list, which looks asymmetric if the system is open ($p < 1$). Simulations are averages over 10^5 realizations (with $T = 10$) in a system of size $N = 10^2$.

is open ($p < 1$). Thus, in closed systems, the probability of changing rank is larger for elements in the middle of the ranking list, and lower at its extremes. In open systems, the bottom of the ranking list ($r \sim p$) is also unstable.

S4.7 Approximation for rank inertia

Here we derive an approximate, closed expression for the inertia measure S_t^{++} introduced in Section S3.6. Similarly to the case of empirical data, in the model we define the matrix element S_t^{ij} as the probability that an element in region i will move to region j after a time $t = s\Delta r$. Regions i, j are divided by an arbitrary threshold $c = \Delta r/p, \ldots, 1$ into the top (+) of the ranking ($r = \Delta r, \ldots, cp$) and the bottom (−) of the ranking ($r = cp + \Delta r, \ldots, p$). Inertia S_t^{++} is then given by

$$S_t^{++} = \sum_{x=\Delta r}^{cp} P_{x,t} = (1 - \nu\Delta r)^s \sum_{x=\Delta r}^{cp} Q_{x,t}. \tag{S42}$$

with $r = \Delta r, \ldots, cp$. The sum in the right hand side of Eq. (S42) is a particular case of the partial cumulative $Q_{[a,b],t} = \sum_{x=a}^{b} Q_{x,t}$ we introduced in Section S4.4, with $a = \Delta r$ and $b = cp$. We can solve directly the associated recurrence relation [Eq. (S25)] with initial condition $Q_{[\Delta r, cp],0} = 1$ using simple formulas for geometric series. Substituting the solution into Eq. (S42) we finally obtain

$$S_t^{++} \simeq e^{-\nu t} \left[cp + (1 - cp) e^{-\tau t} \right], \tag{S43}$$

for $t = s\Delta r$ and sufficiently large N. Eq. (S43) is an analytical approximation for the probability that elements will stay in the top of the ranking across arbitrarily long times, according to our model. Inertia S_t^{++} has an exponential decay with time t, regulated by both τ and ν, which matches with numerical

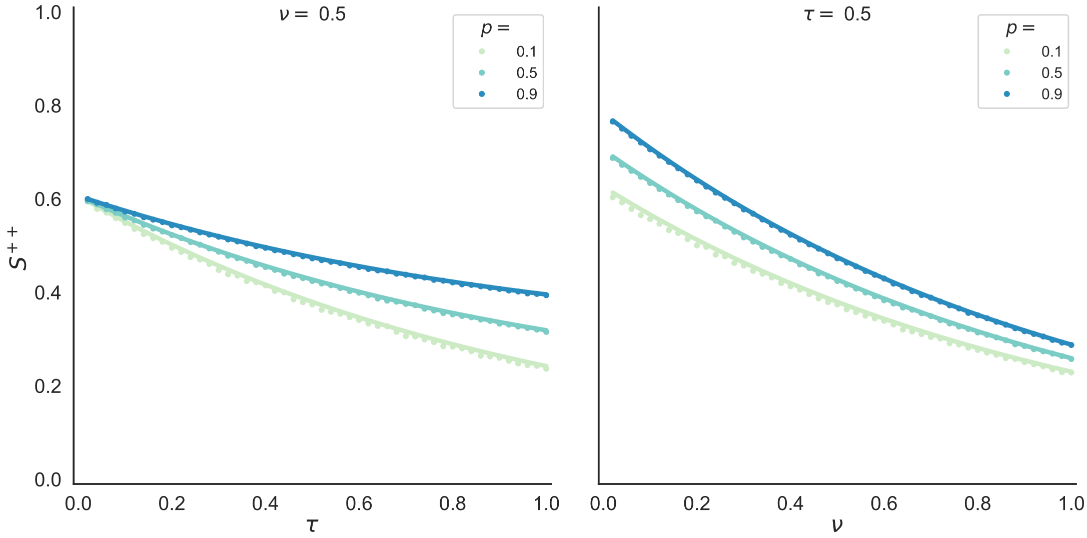

Figure S15. Rank inertia in model. Inertia S^{++} as a function of τ for fixed ν **(left)**, and as a function of ν for fixed τ **(right)**, in both numerical simulations of the model (dots) and the analytical approximation of Eq. (S43) (lines) for varying ranking list size p, calculated for time $t = 1$ ($s = N$) and threshold $c = 0.5$. Inertia decreases as both τ and ν increase, since the displacement/replacement dynamics takes elements out of the top of the ranking list. A relatively longer ranking list (large p) makes it less likely that elements leave the top of the ranking list. Simulations are averages over 10^2 realizations (with $T = 10$) in a system of size $N = 10^4$.

simulations of the model. Denoting $S^{++} \equiv S_t^{++}$ for $t = 1$ (i.e. $s = N$), we see that inertia decreases as τ and ν increase (since there are more displacements and replacements of elements), and conversely, S^{++} is larger for a ranking list of longer size p (Fig. S15). Eq. (S43) captures the decay of inertia with lag in empirical data, although with some deviations (see Fig. S7).

Rank flux F [Eq. (S27)] and inertia S^{++} [Eq. (S43) for $t = 1$] have similar shapes, since they both measure the chance that elements leave, or stay, in (part of) the ranking list. Combining these equations gives a linear constitutive relation between flux and inertia,

$$S^{++} = \beta(1 - F), \qquad \beta = \frac{cp + (1 - cp)e^{-\tau}}{p + (1 - p)e^{-\tau}}. \tag{S44}$$

Intuitively, the probability of staying in the top of the ranking list *decreases linearly* with the flux of elements out of (or into) the ranking list. For $p = 0$ (a closed system) we get a slope $\beta = 1$, while for $p = 1$ and large τ (an open system with displacement dynamics) we get a slope regulated by the inertia threshold parameter, $\beta \simeq c$. This defines a region in (F, S^{++})-space where the model can reproduce pairs of (F, S^{++}) values coming from the empirical datasets, an area that becomes broader as c decreases.

S5 Fitting data with model

Here we describe the process of fitting the rank measures of all considered datasets with the minimal model of rank dynamics introduced in Section S4. First, we set N_0, T and the time series N_t with the empirical values of each dataset listed in Table S1. As described in Section S1 and Section S4.1, we classify ranking lists as closed if $N_t = N_0$ (for all t) and as open otherwise. Then, closed ranking lists have flux $F_t = 0$, out-flux $F_R^- = 0$, and turnover $o_t = 1$ for all t and R, while open ranking lists have

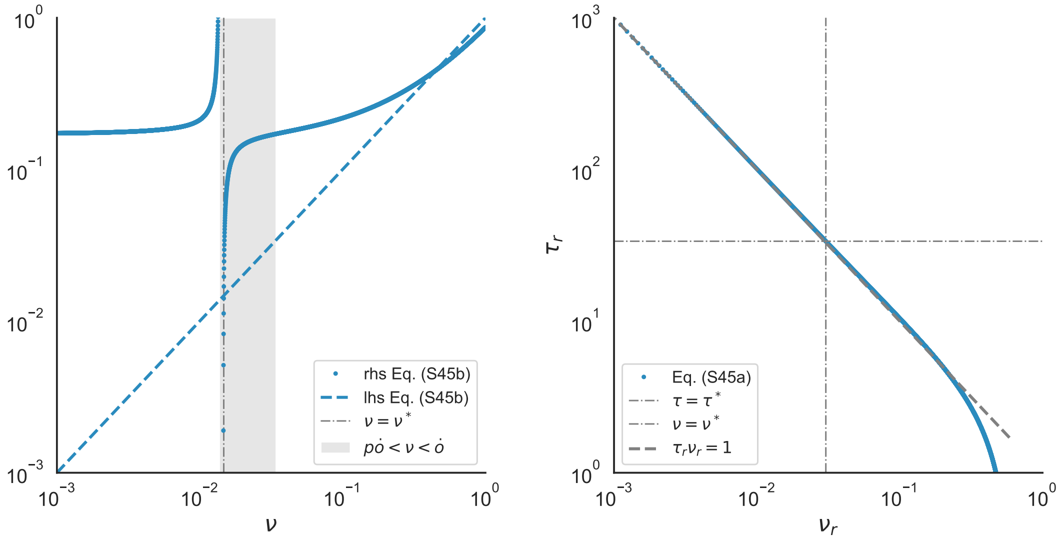

Figure S16. Fitting data with model in open ranking lists. (left) Graphical solution of the transcendental equation for ν defined by Eq. (S45). The right hand side (rhs) of Eq. (S45b) has a singularity at $\nu = p\dot{o}$ and crosses the identity in the left hand side (lhs) of Eq. (S45b) in two fixed points. We only consider the solution ν^* in the interval that ensures $\tau > 0$ in Eq. (S45a) (shaded area). **(right)** Eq. (S45a) for rescaled τ_r and ν_r [see Eq. (S47)], which allows us to find τ^*. The solution (τ^*, ν^*) collapses to the universal curve $\tau_r \nu_r = 1$ when rescaling by the empirical values of ranking list size p, flux F, and mean turnover rate $\dot{o}$. Data corresponds to the Scientists dataset [4] (see Table S1 and Section S2), but the graphical solution is qualitatively the same for all open ranking lists.

non-zero flux and out-flux, and turnover that increases in time. We find five strictly closed systems [Metro stations (London and Mexico), Cities (GB), and Regions JP (quake mag and quakes)], while the rest have varying degrees of turnover, quantified as a pair of scalars by the mean flux $F \in [0, 1]$ and the mean turnover rate $\dot{o} \in [0, 1]$. In terms of model parameters, for closed ranking lists we choose $N = N_0$ (i.e. $p = 1$) and $\nu = 0$, so τ is the only free parameter. For open ranking lists we take $N = N_{T-1}$ (i.e. $p = N_0/N_{T-1}$), so both τ and ν are free parameters.

We consider open ranking lists first. From Section S4.4 and Section S4.5 we have explicit expressions for F [Eq. (S27)] and $\dot{o}$ [Eq. (S40)] in the model, which become more accurate as N and T increase. These relations lead to a non-linear system of equations for τ and ν,

$$\tau = \nu \frac{\dot{o} - \nu}{\nu - p\dot{o}}, \tag{S45a}$$

$$\nu = \ln\left(\frac{p + (1-p)e^{-\tau}}{1 - F}\right), \tag{S45b}$$

determined by the specific values of ranking list size, flux, and turnover of each dataset. Solving for ν (or τ) leads to a transcendental equation in ν (or τ) with no explicit solution. Still, we can gain insight by applying a graphical method and find the solution (τ^*, ν^*) numerically (Fig. S16). From Eq. (S45a) we first note that, in order to ensure $\tau > 0$, ν has to be in the interval $p\dot{o} < \nu < \dot{o}$. Since $\dot{o}$ is relatively low for all studied datasets (less than ~ 0.3; see Table S2), we only need to consider small ν values in the fitting process. Within this range we find a single solution ν^* by graphically equating the two sides of Eq. (S45b) (left panel in Fig. S16). This value can be inserted into Eq. (S45a) to find the remaining solution τ^* (right panel in Fig. S16).

The fitting process for closed ranking lists is, in turn, quite straightforward. Closed ranking lists

Dataset	Data measure F	$\dot{o}$	S^{++}	Model parameter p	τ	ν	ν/ℓ (days^{-1})
**Society**							
GitHub repositories [1]	0.6461	0.1287	0.4339	0.0106	1.0000	0.0015	0.001550
The Guardian readers (recc) [2]	0.7996	0.2085	0.1288	0.0258	1.0000	0.0061	0.006102
The Guardian readers (comm) [2]	0.6118	0.1283	0.3764	0.0413	1.0000	0.0060	0.006027
Enron emails [3]	0.5215	0.2158	0.5394	0.0443	0.7747	0.0129	0.001850
Scientists [4, 5]	0.1578	0.0343	0.8083	0.3982	0.2767	0.0147	0.000040
Universities [6]	0.0477	0.0308	0.9532	0.7143	0.0907	0.0238	0.000065
**Languages**							
Russian [8–11]	0.1796	0.0331	0.8436	0.1262	0.2244	0.0048	0.000013
Spanish [8–11]	0.1603	0.0245	0.8585	0.1361	0.2006	0.0037	0.000010
German [8–11]	0.1605	0.0292	0.8677	0.1159	0.1957	0.0039	0.000011
French [8–11]	0.1849	0.0255	0.8338	0.0967	0.2238	0.0027	0.000008
Italian [8–11]	0.1489	0.0234	0.8684	0.1495	0.1875	0.0039	0.000011
English [8–11]	0.1272	0.0181	0.8928	0.1426	0.1566	0.0029	0.000008
**Economics**							
Companies [12]	0.0734	0.0558	0.9323	0.2639	0.0689	0.0260	0.000071
Countries [13–15]	0.0372	0.0084	0.8967	0.7122	0.1137	0.0061	0.000017
**Infrastructure**							
Cities (RU) [16]	0.1221	0.1073	0.8824	0.5711	0.1230	0.0793	0.000018
Metro stations (London) [17]	0.0000	0.0000	0.9207	1.0000	0.1728	0.0000	0.000000
Cities (GB) [18]	0.0000	0.0000	0.9517	1.0000	0.1015	0.0000	0.000000
Metro stations (Mexico)	0.0000	0.0000	0.9100	1.0000	0.1984	0.0000	0.000000
**Nature**							
Hyenas [19]	0.3023	0.2748	0.5289	0.1419	0.3213	0.0911	0.000249
Regions JP (quake mag) [20, 21]	0.0000	0.0000	0.7293	1.0000	0.7797	0.0000	0.000000
Regions JP (quakes) [20, 21]	0.0000	0.0000	0.7625	1.0000	0.6444	0.0000	0.000000
**Sports**							
Chess players (male) [22]	0.0106	0.0051	0.9877	0.8148	0.0354	0.0042	0.000138
Chess players (female) [22]	0.0099	0.0068	0.9929	0.7667	0.0193	0.0055	0.000182
Poker players [24]	0.0377	0.0203	0.9714	0.1832	0.0402	0.0058	0.000824
Tennis players [26]	0.0112	0.0050	0.9916	0.3338	0.0137	0.0021	0.000302
Golf players [28]	0.0058	0.0028	0.9904	0.3166	0.0068	0.0012	0.000168
Football scorers [30]	0.1340	0.0960	0.9091	0.1669	0.1377	0.0305	0.004339
NASCAR drivers (Busch) [32]	0.3648	0.2392	0.6348	0.1124	0.4744	0.0455	0.000124
NASCAR drivers (Winston Cup) [32]	0.1865	0.1306	0.8145	0.1838	0.2052	0.0422	0.000115
National football teams [33]	0.0031	0.0007	0.9728	0.9524	0.0532	0.0000	0.000000

Table S2. Data measures and fitted model parameters. Values of empirical measures used in the fitting process (mean flux F, mean turnover rate $\dot{o}$, and inertia S^{++}) and of fitted model parameters (relative ranking list size p, displacement probability τ, and replacement probability ν) for all considered datasets (see Table S1 and Section S2). For open ranking lists, we fit the model to empirical data by setting $N = N_{T-1}$ (i.e. $p = N_0/N_{T-1}$, see Section S4.1) and by computing τ and ν numerically from Eq. (S45) in terms of F and $\dot{o}$ (Fig. S16). For closed ranking lists, we set $N = N_0$ and $\nu = 0$, and obtain τ explicitly from Eq. (S46) in terms of S^{++} (for time $t = 1$ and threshold $c = 0.5$). We also show the rate of element replacement ν/ℓ, with ℓ the real time between observations (see Table S1). Datasets are classified by an (arbitrary) system type based on the nature of the elements in the ranking list.

have trivial values of flux and turnover, so we cannot use Eq. (S45) for fitting. Instead we consider the explicit expression of inertia S^{++} (for time $t = 1$) derived in Section S4.7. S^{++} is reminiscent of F in the sense that it measures the flux of elements out of (into) the top of the ranking list into (from) the

bottom, where top and bottom are arbitrarily separated by the threshold c. Solving for τ in Eq. (S44) gives

$$\tau = \ln\left(\frac{1-cp}{S^{++}-cp}\right), \qquad F=0, \tag{S46}$$

which allows us to find τ for all closed ranking lists considered.

Table S2 summarizes the results of the fitting process described above. Our minimal model of rank dynamics fits most rank measures of many datasets (see Figs. S3-S7). In the model, rank flux F_t is a constant over time that recovers the empirical mean with high precision, apart from fluctuations (Fig. S3). Out-flux F_R^- is relatively constant for most R values, and in open systems has a sharp increase for large R. This is only a good fit for relatively closed ranking lists, since in very open systems out-flux is a more gradually increasing function of R (Fig. S4). Turnover o_t is concave and monotonically increasing in time, which follows empirical data (Fig. S5). Rank change C recovers the asymmetry of most open systems (low values only in the top of the ranking list) and the rough symmetry in closed systems (low values in both the top and bottom of the ranking list), although empirical closed systems are not as symmetric as the model predicts (Fig. S6). Inertia S_t^{++} is a decaying function of time t, in agreement with several datasets. Even if the difference in qualitative behavior between open and closed ranking lists is less pronounced for S_t^{++} than for other rank measures, we still see that inertia tends to decay faster in open systems (Fig. S7).

S5.1 Dynamical regimes of open ranking lists

Having explicit expressions in Eq. (S45) lets us further analyze the solution (τ^*, ν^*). We introduce the rescaled parameters

$$\tau_r = \frac{\tau}{p(1-p)\dot{o}}, \quad \text{and} \quad \nu_r = \frac{\nu - p\dot{o}}{\dot{o}}, \tag{S47}$$

which allows us to rewrite Eq. (S45a) for sufficiently small ν (relative to p and $1-p$),

$$\tau_r \nu_r \simeq 1. \tag{S48}$$

Eq. (S48) shows that, when fitting the model to datasets with given values of p, F, and $\dot{o}$, the probabilities τ and ν can be rescaled to collapse into a universal curve, a power law with exponent 1 (Fig. S16). In order to reproduce the dynamics of empirical ranking lists, is it enough to consider an inverse relationship between the rates of displacement and replacement of elements in the model, meaning that the considered datasets have either an active dynamics of Lévy flights and diffusion plus a few replacements, or less displacements with a larger exchange of elements. This makes the fitting process effectively one-dimensional across open ranking lists, even if the model can be studied for arbitrary τ and ν.

The universal curve in Eq. (S48) displays three regimes in the dynamics of open ranking lists, as measured by the average probabilities that, between consecutive observations in the data (i.e. between times t and $t+1$), an element performs either a Lévy walk,

$$W_{\text{levy}} = e^{-\nu}(1 - e^{-\tau}), \tag{S49}$$

changes rank by diffusion

$$W_{\text{diff}} = e^{-\nu} e^{-\tau}, \tag{S50}$$

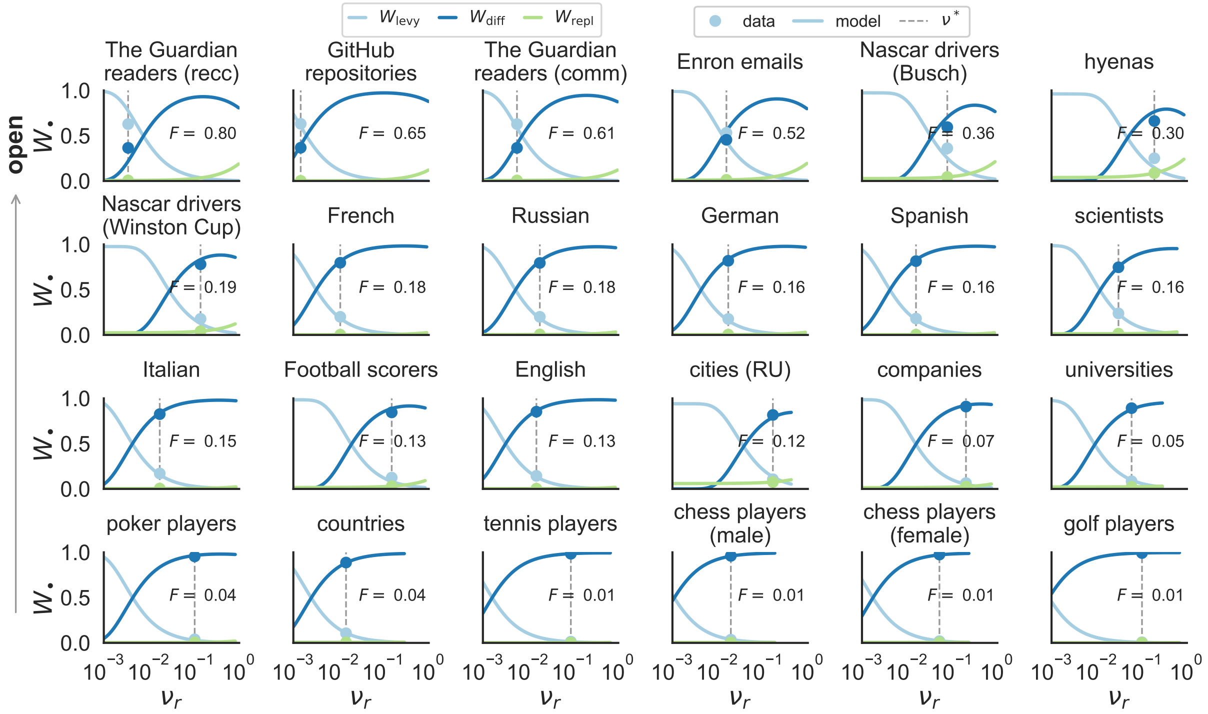

Figure S17. Regimes of dynamical behavior in open ranking lists. Average probability that an element changes rank by Lévy walk ($W_{\rm levy}$), diffusion ($W_{\rm diff}$), or is replaced ($W_{\rm repl}$) between consecutive observations in the data. Probabilities shown both for all datasets with $\dot{o} > 10^{-3}$ (dots), and for the model moving along the curve $\tau_r \nu_r = 1$ [Eq. (S48)] with the same p and $\dot{o}$ as the data (lines). Datasets are ordered from most open (upper row) to least open (lower row) according to mean rank flux F [Eq. (S2)]. The model reveals a crossover in empirical ranking lists between Lévy walk ($W_{\rm levy} > W_{\rm diff}, W_{\rm repl}$) and diffusion ($W_{\rm diff} > W_{\rm levy}, W_{\rm repl}$) regimes. Although not seen in data, there is also a third regime driven by replacement ($W_{\rm repl} > W_{\rm levy}, W_{\rm diff}$).

or is replaced,

$$W_{\rm repl} = 1 - e^{-\nu}, \tag{S51}$$

where $W_{\rm levy} + W_{\rm diff} + W_{\rm repl} = 1$. In the most open systems we study, elements mostly change rank via long jumps, forming a Lévy walk regime where $W_{\rm levy} > W_{\rm diff}, W_{\rm repl}$ (Fig. S17). Here, long-range rank changes take elements in and out of a small ranking list within a big system (low p), thus generating large mean flux F. The rest of the datasets belong to a diffusion regime with $W_{\rm diff} > W_{\rm levy}, W_{\rm repl}$. A local, diffusive rank dynamics is the result of elements smoothly changing their scores and overcoming their neighbors in rank space. Although not observed in empirical data, the model predicts a third regime dominated by replacement ($W_{\rm repl} > W_{\rm levy}, W_{\rm diff}$). The curves in Fig. S17 show how close a ranking list might be to a change of regime. If a dataset has values of p and $\dot{o}$ that make it fall close to the boundaries of these regimes, small changes in the parameters regulating the displacement (τ) and replacement (ν) can change the dynamical regime of the ranking list.

S5.2 Fluctuations, estimation bias, and goodness of fit

The relationship between rescaled parameters τ_r and ν_r in Eq. (S48) is a consequence of fitting average values of flux F and turnover rate $\dot{o}$ in empirical data with asymptotic approximations coming from the model. Here we investigate the fluctuations around such average values arising in numerical simulations of the model, which reveal a small but nonzero bias in the estimation of τ. Despite this bias, simulations

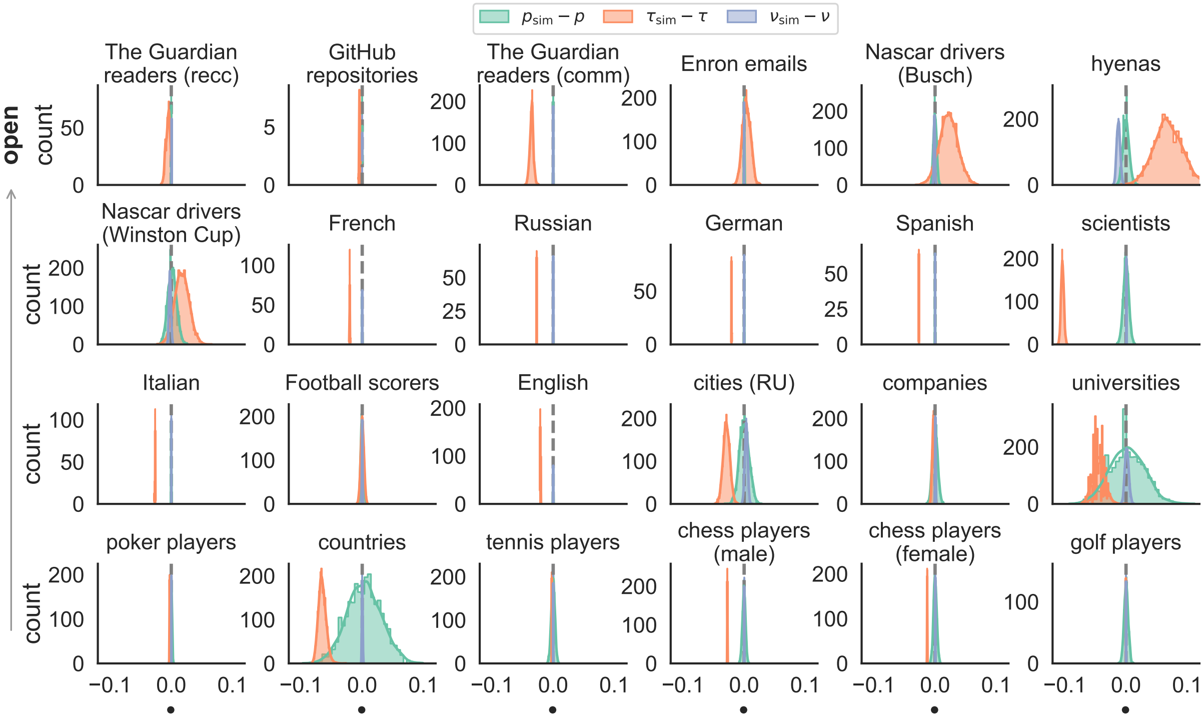

Figure S18. Model fluctuations and estimation bias in open ranking lists. Distributions of bootstrapped parameter differences $\Delta p = p_{\rm sim} - p$, $\Delta\tau = \tau_{\rm sim} - \tau$, and $\Delta\nu = \nu_{\rm sim} - \nu$ between fitted parameters p, τ, and ν of a dataset, and the corresponding $p_{\rm sim}$, $\tau_{\rm sim}$, and $\nu_{\rm sim}$ when fitting the model to itself. Distributions are plotted as binned counts (histograms) and kernel density estimates (continuous lines). The dashed line at 0 indicates perfect agreement between fitted parameters and bootstrapped estimates. Number of simulations varies from 30 to 2500 per system. Datasets (with $\dot{o} > 10^{-3}$) are ordered from most open (upper row) to least open (lower row) according to mean rank flux F [Eq. (S2)].

recover the empirical values of system-wide quantities (F, $\dot{o}$, and inertia S^{++}), showcasing the ability of the model to reproduce rank dynamics in data. We further quantify the goodness of fit by showing that, for most systems considered, data is closer to the universal curve [Eq. (S48)] than model simulations.

We start by taking a dataset with given values of T, $p = N_0/N_{T-1}$, and fitted parameters τ and ν (see Tables S1-S2). We first run a large number of realizations of the model with the same values of N_0, T, τ, and ν, plus an arbitrary but smaller system size $N_{\rm sim} < N_{T-1}$, leading to a certain number of elements ever seen in the ranking list of each simulation, which we denote by $N_{T-1,{\rm sim}}$. Since the model is stochastic, $N_{T-1,{\rm sim}}$ varies across realizations, with average $\langle N_{T-1,{\rm sim}} \rangle$. We choose the $N_{\rm sim}$ that minimizes the quantity $|N_{T-1} - \langle N_{T-1,{\rm sim}} \rangle|$, ensuring that $p_{\rm sim} = N_0/N_{T-1,{\rm sim}}$ is close to the empirical p, beyond fluctuations. With $N_{\rm sim}$ fixed, we again run a large number of realizations of the model with the same values of N_0, T, τ, and ν. Each model realization will have estimated values of flux $F_{\rm sim}$, turnover rate $\dot{o}_{\rm sim}$, and inertia $S^{++}_{\rm sim}$ similar to those in the original dataset (apart from fluctuations), which we use to fit the model to itself [via Eq. (S45) or Eq. (S46)] and obtain estimated parameters $\tau_{\rm sim}$ and $\nu_{\rm sim}$. This constitutes a parametric bootstrap of the model, allowing us to explore expected variations in the fitted parameters τ and ν.

We compare the distributions of $p_{\rm sim}$, $\tau_{\rm sim}$, and $\nu_{\rm sim}$ over model simulations with the fitted parameters p, τ, and ν of open ranking lists by calculating the differences $\Delta p = p_{\rm sim} - p$, $\Delta\tau = \tau_{\rm sim} - \tau$, and $\Delta\nu = \nu_{\rm sim} - \nu$ (Fig. S18). We obtain $\langle \Delta p \rangle \simeq \langle \Delta\nu \rangle \simeq 0$ for all datasets (apart from hyenas, where ν

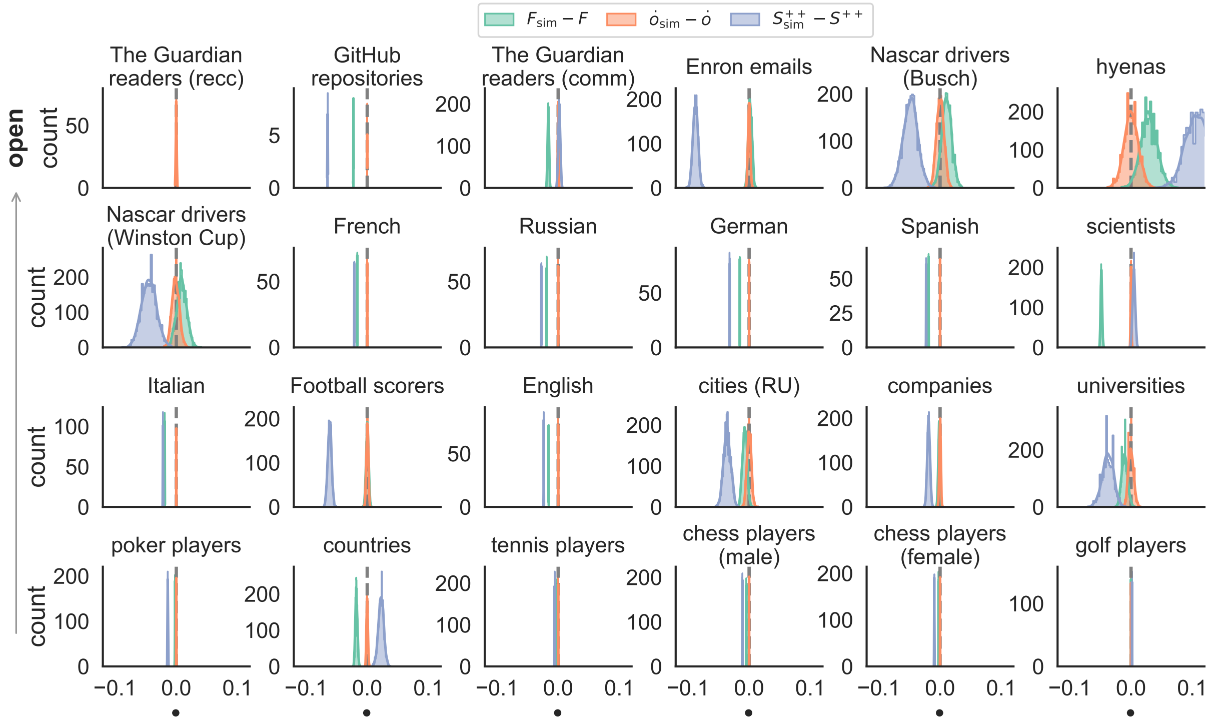

Figure S19. Simulations recover properties of open ranking lists. Distributions of bootstrapped differences $\Delta F = F_{\text{sim}} - F$, $\Delta \dot{o} = \dot{o}_{\text{sim}} - \dot{o}$, and $\Delta S^{++} = S^{++}_{\text{sim}} - S^{++}$ between flux F, turnover rate $\dot{o}$, and inertia S^{++} in a dataset, and the corresponding F_{sim}, $\dot{o}_{\text{sim}}$, and S^{++}_{sim} in simulations of the model with fitted model parameters (see Tables S1-S2). Distributions are plotted as binned counts (histograms) and kernel density estimates (continuous lines). The dashed line at 0 indicates perfect agreement between empirical properties and bootstrapped estimates. Number of simulations varies from 30 to 2500 per system. Datasets (with $\dot{o} > 10^{-3}$) are ordered from most open (upper row) to least open (lower row) according to mean rank flux F [Eq. (S2)].

is slightly underestimated), meaning that the fitting process consistently recovers the values of p and ν in simulations, without bias. Fluctuations around $\langle \Delta p \rangle$, $\langle \Delta \tau \rangle$, and $\langle \Delta \nu \rangle$ (measured by the spread of their associated distributions) are only noticeable for some of the smallest datasets (Enron emails, hyenas, Nascar drivers, cities [RU], universities, and countries). Even when accounting for fluctuations, we find a small bias in τ ($|\langle \Delta \tau \rangle| < 0.1$) for several datasets [The Guardian readers (comm), hyenas, languages, scientists, cities (RU), universities, countries, and chess], indicating that the fitting process systematically under- ($\Delta \tau < 0$) or over- ($\Delta \tau > 0$) estimates the displacement probability in simulations, particularly in systems with low N_{T-1} or T.

Despite the small bias in τ (Fig. S18), numerical simulations with fitted model parameters recover aggregate properties of empirical data, which we quantify via the differences $\Delta F = F_{\text{sim}} - F$, $\Delta \dot{o} = \dot{o}_{\text{sim}} - \dot{o}$, and $\Delta S^{++} = S^{++}_{\text{sim}} - S^{++}$ (Fig. S19). Turnover rate is perfectly reproduced in all datasets ($\langle \Delta \dot{o} \rangle \simeq 0$), apart from fluctuations, while flux has a small disagreement ($|\langle \Delta F \rangle| < 0.1$) for some systems [GitHub repositories, The Guardian readers (comm), hyenas, languages, scientists, and countries]. Simulations even recover, to some extent, quantities not involved in the fitting of open ranking lists: inertia is perfectly reproduced ($\langle \Delta S^{++} \rangle \simeq 0$) in The Guardian, scientists, tennis, chess, and golf datasets, and has a small disagreement ($|\langle \Delta S^{++} \rangle| < 0.1$) for the rest.

In order to further quantify the goodness of fit, we explore how the rescaled fitted parameters and bootstrapped estimates fall around the universal curve of Eq. (S48). For a dataset with given values of

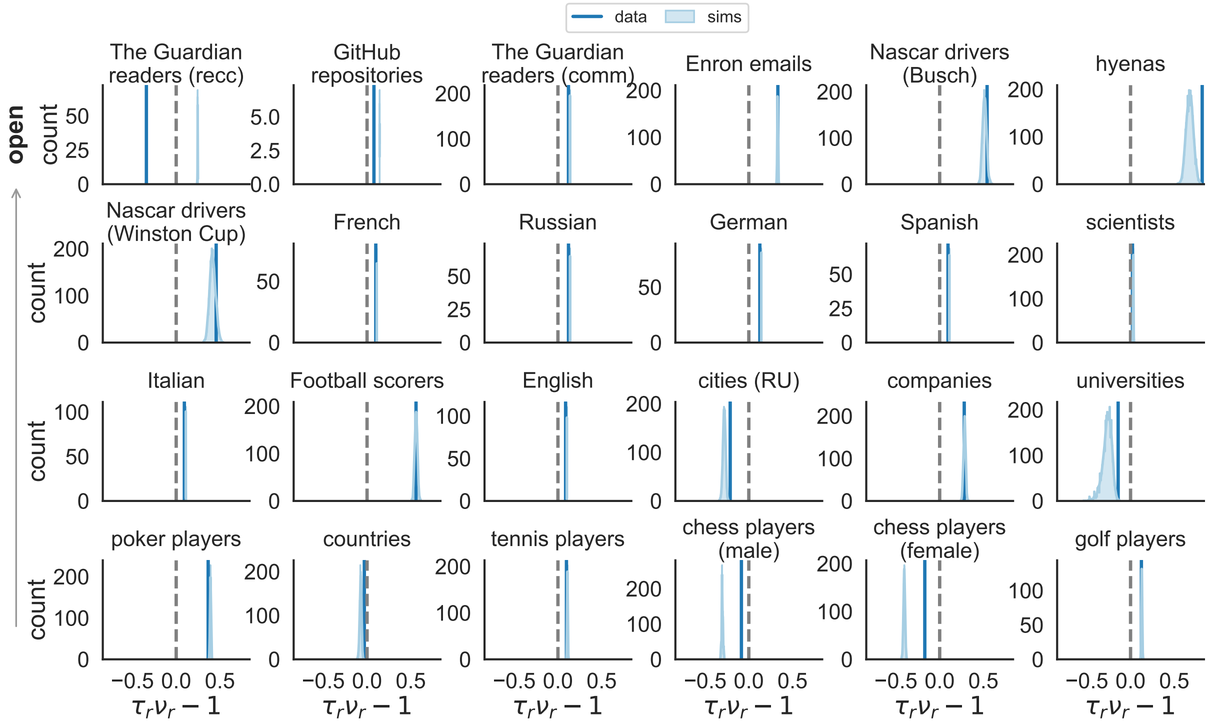

Figure S20. Distance to universal curve in open ranking lists. Distribution of relative difference $\tau_r \nu_r - 1$ between rescaled parameters (τ_r and ν_r) and universal curve [$\tau_r \nu_r = 1$; see Eq. (S48)], both for the fitted parameters τ and ν of a dataset (data; shown as vertical line), and for the corresponding τ_{sim} and ν_{sim} when fitting the model to itself (sims). Distributions are plotted as binned counts (histograms) and kernel density estimates (continuous lines). The dashed line at 0 indicates perfect agreement between fitted parameters / bootstrapped estimates and the universal curve in Eq. (S48). Number of simulations varies from 30 to 2500 per system. Datasets (with $\dot{o} > 10^{-3}$) are ordered from most open (upper row) to least open (lower row) according to mean rank flux F [Eq. (S2)].

p, $\dot{o}$, and fit τ and ν, the rescaled parameters τ_r and ν_r in Eq. (S47) have the same relative difference from the expected universal behavior ($\tau_r \nu_r = 1$) alongside either axis,

$$\frac{\tau_r - 1/\nu_r}{1/\nu_r} = \frac{\nu_r - 1/\tau_r}{1/\tau_r} = \tau_r \nu_r - 1. \tag{S52}$$

We measure $\tau_r \nu_r - 1$ for all datasets, as well as the distribution of the corresponding quantity $\tau_r^{\text{sim}} \nu_r^{\text{sim}} - 1$ over model simulations, calculated with p_{sim}, $\dot{o}_{\text{sim}}$, and the bootstrapped fit τ_{sim} and ν_{sim} [8] (Fig. S20). We do not expect rescaled parameters to follow the universal curve perfectly, due to the approximations used to derive F and $\dot{o}$ in the model [Eq. (S27) and Eq. (S40); see Sections S4.4-S4.5], as well as the assumption of small ν in the derivation of Eq. (S48) itself (see Section S5.1 and Fig. S16). Still, in all datasets we find good agreement for fitted model parameters ($|\tau_r \nu_r - 1| < 0.5$) and bootstrapped estimates ($|\langle \tau_r^{\text{sim}} \nu_r^{\text{sim}} \rangle - 1| < 0.5$). For most systems, the dataset has τ_r and ν_r closer to the universal curve than most simulations of the model itself, suggesting than empirical ranking lists follow Eq. (S48). Exceptions are The Guardian readers (recc), Enron emails, Nascar drivers (Busch), and hyenas. We consider that these systems do not follow the proposed universal behavior either because of small sample size (leading to data noise), or due to a systematic failure of the model to recover their dynamics.

Finally, it's worth noting that it would be ideal to have a more formal statistical approach to model

[8] Explicitly, $\tau_r^{\text{sim}} = \frac{\tau_{\text{sim}}}{p_{\text{sim}}(1-p_{\text{sim}})\dot{o}_{\text{sim}}}$ and $\nu_r^{\text{sim}} = \frac{\nu_{\text{sim}} - p_{\text{sim}}\dot{o}_{\text{sim}}}{\dot{o}_{\text{sim}}}$, following Eq. (S47).

fitting than the process used here. It is indeed possible to derive maximum likelihood equations for two probability distributions: (i) The distribution of the time t an element stays in the ranking list [the product $\dot{o}S_t^{++}$ of mean turnover rate and time-dependent rank inertia according to Eq. (S40) and Eq. (S43)]; and (ii) the probability of element displacement in Eq. (S21). The maximum likelihood equations (for τ and ν) coming from maximizing the log-likelihoods of these two distributions are trascendental and cannot be solved analytically. We can, however, solve the equations numerically and compare their solutions to the fitted τ and ν from our 'mean-field' fitting process. For most datasets, mean-field parameter estimates lead to lower mean-squared-error deviations between the fitted model and several measures in the data (rank flux, turnover, out-flux, change probability, rank diversity, and inertia). This means that our fitting process is able to recover aggregate features of empirical ranking data, despite it being based on asymptotic approximations of mean behavior and showing a small bias in estimating τ. As added value, the mean-field fitting process is more analytically tractable, computationally faster to obtain, and provides insight on the balance between amounts of element displacement and replacement seen in data.

S5.3 Effect of subsampling

Our model of ranking dynamics suggests that empirical ranking lists belong to one of several dynamical regimes constrained by the universal curve in Eq. (S48). Here we explore the role of sampling rate (related to the time between observations of the ranking, see ℓ in Table S1) in the regime occupied by a system. We show that subsampling observations typically makes systems go downwards along the universal curve (right panel in Fig. S16 and Fig. 3 in main text), moving, for example, from a regime with many Lévy walks to one more driven by diffusion.

For a synthetic or empirical system with T observations, we implement subsampling by only considering the ranking list at times $t = 0, k, 2k, \ldots, T_{\text{eff}} - 1$ and discarding the rest of the data, with $k = 1, 2, \ldots, T-1$ a tunable parameter. The subsampling process decreases the number of observations to $T_{\text{eff}} = \lceil T/k \rceil \leq T$, such that $k = 1$ recovers the original dataset ($T_{\text{eff}} = T$) and $k = T - 1$ corresponds to the most subsampled system possible ($T_{\text{eff}} = 2$). We then calculate the (k-dependent) subsampled rank flux F_k, turnover rate $\dot{o}_k$, and inertia S_k^{++}, and compare them with their values F, $\dot{o}$, and S^{++} in the original ranking list. Finally, we fit the model to the subsampled data [via Eq. (S45) or Eq. (S46)] for each k value, obtaining the subsampled relative list size p_k, displacement τ_k^* and replacement ν_k^*, in contrast with the original fit p, τ^*, and ν^*.

We can gain some insight on the role of subsampling by first analyzing synthetic ranking lists coming from the model itself. Consider a model dynamics (with parameters N, τ, and ν) and its subsampled version (with effective parameters τ_k and ν_k for given k) (Fig. S21). The probability that an element is not replaced between two subsampled observations of the original dynamics, $(1 - \nu\Delta r)^{kN}$, must be equal to the probability of not being replaced between consecutive observations of the subsampled dynamics, $(1 - \nu_k \Delta r)^N$. A similar argument applies to τ and τ_k. Taking the limit $N \to \infty$ and the first-order power series of the exponential, we obtain

$$\tau_k = 1 - (1 - \tau)^k, \tag{S53a}$$

$$\nu_k = 1 - (1 - \nu)^k, \tag{S53b}$$

meaning that subsampling increases the rates of displacement and replacement in the model, almost linearly for small enough τ and ν. Eq. (S53) allows us to write approximate analytical expressions for the

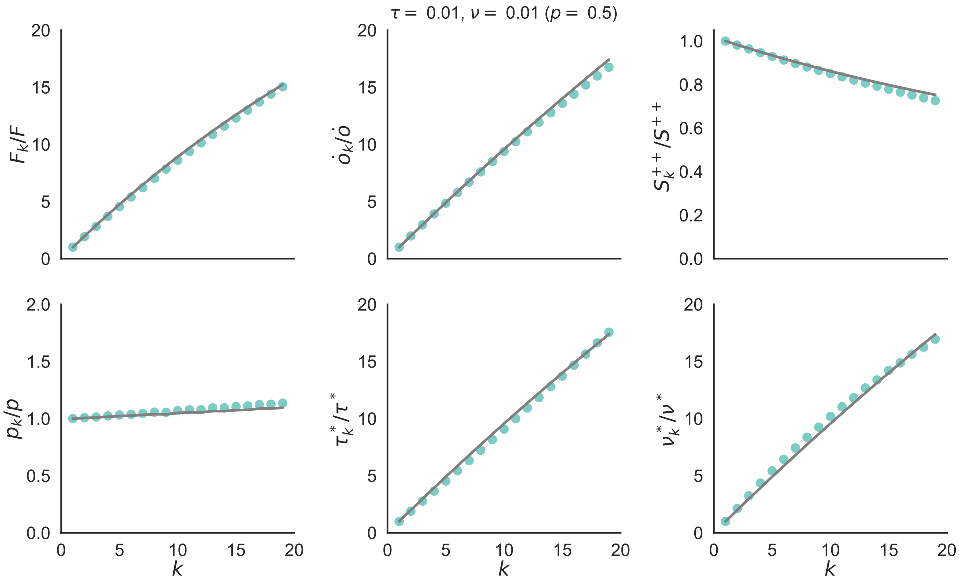

Figure S21. Subsampling in model. Rank flux F_k, turnover rate $\dot{o}_k$, and inertia S_k^{++} (for threshold $c = 0.5$), as well as fitted parameters p_k, τ_k^* and ν_k^* as a function of subsampling parameter k, relative to their original values (F, $\dot{o}$, S^{++}, p, τ^*, and ν^*) for fixed τ, ν, and p. Results are both numerical simulations of the model (dots) and their corresponding analytical approximations (lines), with τ_k and ν_k given by Eq. (S53). Subsampling increases the rates of displacement and replacement, leading to larger flux and turnover, and lower inertia. We only show k values such that $T_{\text{eff}}/T > 0.05$. Simulations are averages over 10 realizations (with $T = 10^3$) in a system of size $N = 10^3$.

subsampled rank flux F_k, turnover rate $\dot{o}_k$, and inertia S_k^{++} by replacing (τ, ν) with (τ_k, ν_k) in Eq. (S27), Eq. (S38), and Eq. (S43), respectively. As k grows, larger rates of displacement and replacement lead to an increase in flux and turnover, and a corresponding lower probability to stay in the top of the ranking. Since some elements enter and leave the ranking list between subsampled observations, the number of elements ever seen in the list tends to decrease, leading to a slightly larger p_k (Fig. S21). We note that the approximation of Eq. (S53) might become less accurate for some parameter values and as k increases.

We perform the same subsampling process for all open empirical ranking lists, and further calculate the rescaled parameters τ_r and ν_r for varying k [by using Eq. (S47) with the subsampled quantities τ_k^* ν_k^*, p_k, and $\dot{o}_k$ instead of their original values] (Fig. S22). As subsampling increases, most datasets go downwards along the universal curve of Eq. (S48) in (ν_r, τ_r)-space, moving from a regime with a certain amount of Lévy walks to one more driven by diffusion. Some datasets do not follow this trend [Nascar drivers, hyenas, cities [RU], companies, universities], which follows from the condition $\nu_k^* \sim 0$ in Eq. (S48) not being fulfilled for large enough k (see Fig. S21). We also notice some overlap between these datasets and the ones showing both large fluctuations and distance to the universal curve in Section S5.2 (Fig. S18 and Fig. S20), further suggesting that only a few empirical ranking lists have dynamics not captured by the model and its universal curve.

S6 On the heterogeneity of rank dynamics

Our results suggest that rank dynamics are *heterogeneous*: some elements change their rank slower than others, even when the dynamics of the elements themselves (i.e. the scores) is homogeneous. Soccer teams play roughly the same number of games; metro stations are open mostly at the same time of day.

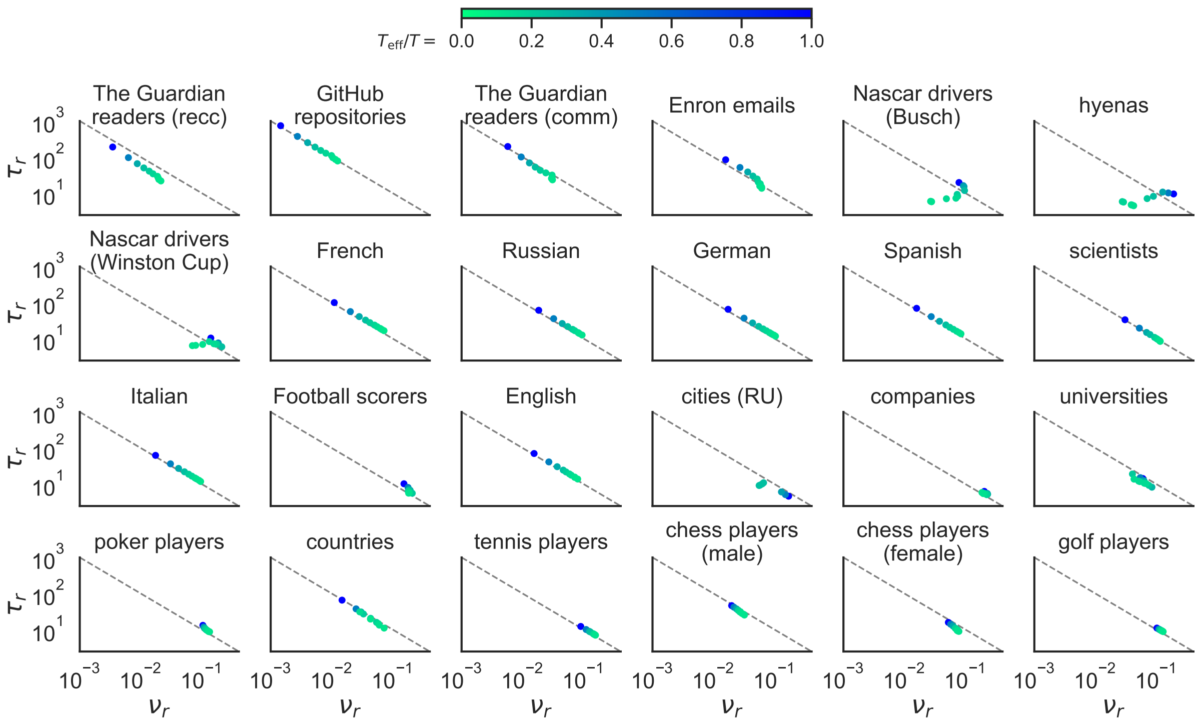

Figure S22. Subsampling in open ranking lists. Rescaled parameters τ_r and ν_r for varying subsampling parameter k, color coded by the effective number of observations $T_{\text{eff}} = \lceil T/k \rceil$ relative to its original value T. As subsampling increases, the fitted model estimates movement along the universal curve from the Lévy to the diffusion regimes. We only use k values such that $T_{\text{eff}}/T > 0.1$. Datasets (with $\dot{o} > 10^{-3}$) are ordered from most open (upper row) to least open (lower row) according to mean rank flux F [Eq. (S2)].

So why are rank dynamics heterogeneous? A possibility is that systems require additional mechanisms for maintaining a homogeneous temporality. Still, are there advantages to such a heterogeneity? When considering open systems in close interaction with their surroundings (and for which the entirety of the associated ranking has a physical meaning, e.g. in the case of a genetic code), there is evolutionary advantage in preserving the functionality of the most essential elements (to maintain robustness [44]), while allowing for fast variability of less crucial components (conferring adaptivity [45]). An understanding of the balance between stability and variability has so far been mostly constrained to critical phase transitions in homogeneous models [46–48]. We pose the following hypothesis: if we assume varying timescales of dynamical behavior according to rank (with elements at the top of a ranking list changing more slowly than the rest), systems would benefit by having robustness and adaptability at the same time, independently of critical parameters: "slower" elements would provide robustness, while "faster" elements would provide adaptivity. The ability of a simple model to reproduce rank dynamics in a wide variety of phenomena, regardless of their domain, suggests that such balanced dynamics can be achieved with random rank change. Thus, complex systems may need much less to be evolvable than previously thought (on the basis of homogeneous timescales only), implying that random variation of natural selection, if heterogeneous, is enough to produce the complex adaptations seen in evolutionary biology and computer science [49]. This hypothesis remains to be explored further, but it might be useful to guide the interpretation of the results presented here.

Supplementary References

[1] List of repositories, GitHub. `https://github.com/explore`. Accessed: 2018-05-29.

[2] The Guardian, UK edition. `https://www.theguardian.com/uk`. Accessed: 2018-07-04.

[3] Enron email dataset, CALO Project. `https://www.cs.cmu.edu/~enron/`. Accessed: 2018-05-29.

[4] R. Sinatra, P. Deville, M. Szell, D. Wang, and A.-L. Barabási. A century of physics. *Nat. Phys.* **11**, 791–797 (2015).

[5] R. Sinatra, D. Wang, P. Deville, C. Song, and A.-L. Barabási. Quantifying the evolution of individual scientific impact. *Science* **354**, aaf5239 (2016).

[6] Statistics 2016, Academic Ranking of World Universities (ARWU). `http://www.shanghairanking.com/ARWU-Statistics-2016.html`. Accessed: 2018-05-29.

[7] About Academic Ranking of World Universities (ARWU). `http://www.shanghairanking.com/aboutarwu.html`. Accessed: 2018-05-29.

[8] Google Books Ngram Viewer. `http://storage.googleapis.com/books/ngrams/books/datasetsv2.html`. Accessed: 2018-05-29.

[9] J.-B. Michel *et al.*, Quantitative analysis of culture using millions of digitized books. *Science* **331**, 176 – 182 (2011).

[10] G. Cocho, J. Flores, C. Gershenson, C. Pineda, and S. Sánchez, Rank diversity of languages: Generic behavior in computational linguistics. *PLoS ONE* **10**, e0121898 (2015).

[11] J. A. Morales *et al.*, Rank dynamics of word usage at multiple scales. *Front. Phys.* **6**, 45 (2018).

[12] Database 1955–2005, Fortune 500. `http://archive.fortune.com/magazines/fortune/fortune500_archive/full/1955/index.html`. Accessed: 2018-05-29.

[13] The Observatory of Economic Complexity. `https://atlas.media.mit.edu/en/`. Accessed: 2018-05-29.

[14] C. A. Hidalgo and R. Hausmann, The building blocks of economic complexity. *Proc. Nat. Acad. Sci USA* **106**, 10570–10575 (2009).

[15] R. Hausmann *et al.*, *The Atlas of Economic Complexity: Mapping Paths to Prosperity* (MIT Press, 2014).

[16] C. Cottineau. A multilevel portrait of shrinking urban Russia. *Espace Populations Sociétés* (online), 2015/3-2016/1 (2016). Accessed: 2018-06-12.

[17] R. Murcio, C. Zhong, E. Manley, and M. Batty, Identifying risk profiles in the London's public transport system. In: *14th International Conference on Computers in Urban Planning and Urban Management* (Boston, MA, 2015).

[18] R. Edwards and M. Batty, City size: Spatial dynamics as temporal flows. *Environ. Plann. A* **48**, 1001–1003 (2016).

[19] A. Ilany, A. S. Booms, and K. E. Holekamp, Topological effects of network structure on long-term social network dynamics in a wild mammal. *Ecol. Lett.* **18**, 687–695 (2015).

[20] Japan University Network Earthquake Catalog. `https://wwweic.eri.u-tokyo.ac.jp/db/junec/index.html`. Accessed: 2018-07-04.

[21] M. Karsai, K. Kaski, A.-L. Barabási, and J. Kertész, Universal features of correlated bursty behaviour. *Sci. Rep.* **2**, 397 (2012).

[22] FRL June 2018, World Chess Federation. `https://ratings.fide.com/download.phtml`. Accessed: 2018-06-12.

[23] Elo rating system, Wikipedia. `https://en.wikipedia.org/wiki/Elo_rating_system`. Accessed: 2018-06-12.

[24] Rankings, Global Poker Index (GPI). `http://www.globalpokerindex.com/rankings/`. Accessed: 2018-06-13.

[25] About the Global Poker Index (GPI). `http://www.globalpokerindex.com/about/`. Accessed: 2018-06-13.

[26] Rankings (singles), Association of Tennis Professionals (ATP) World Tour. `http://www.atpworldtour.com/en/rankings/singles`. Accessed: 2018-06-13.

[27] Rankings (FAQ), Association of Tennis Professionals (ATP) World Tour. `http://www.atpworldtour.com/en/rankings/rankings-faq`. Accessed: 2018-06-13.

[28] Official World Golf Ranking (OWGR). `http://www.owgr.com/ranking`. Accessed: 2018-06-13.

[29] How the ranking system works, Official World Golf Ranking (OWGR). `http://www.owgr.com/about`. Accessed: 2018-06-13.

[30] Ranking top scorers, Football World Rankings. `https://www.clubworldranking.com/ranking-topscorers`. Accessed: 2018-06-13.

[31] How are the Football World Rankings calculated?, Football World Rankings. `https://www.clubworldranking.com/how-are-the-football-world-rankings-calculated`. Accessed: 2018-06-13.

[32] National Association for Stock Car Auto Racing (NASCAR) standings, Entertainment and Sports Programming Network. `http://www.espn.com/racing/standings`. Accessed: 2018-06-13.

[33] World Ranking, Fédération Internationale de Football Association (FIFA). `https://www.fifa.com/fifa-world-ranking/`. Accessed: 2018-06-13.

[34] Men's ranking procedure, Fédération Internationale de Football Association (FIFA). `https://www.fifa.com/fifa-world-ranking/procedure/men.html`. Accessed: 2018-06-13.

[35] J. A. Morales *et al.*, Generic temporal features of performance rankings in sports and games. *EPJ Data Science* **5**, 33 (2016).

[36] S. Sánchez *et al.*, Trajectory stability in the traveling salesman problem. *Complexity* **2018**, 2826082 (2018).

[37] D. Aldous and P. Diaconis, Shuffling cards and stopping times. *Am. Math. Mon.* **93**, 333–348 (1986).

[38] J. M. Kleinberg, Navigation in a small world. *Nature* **406**, 845 (2000).

[39] G. Li *et al.*, Towards design principles for optimal transport networks. *Phys. Rev. Lett.* **104**, 018701 (2010).

[40] C. A. Muirhead, Genetic drift, models of random. *Encyclopedia of Evolutionary Biology*, 136–143 (2016).

[41] T. D. Tran, J. Hofrichter, and J. Jost, An introduction to the mathematical structure of the Wright-Fisher model of population genetics. *Theor. Biosci.* **132**, 73–82 (2013).

[42] L. Chen and D. W. Stroock, The fundamental solution to the Wright-Fisher equation. *SIAM J. Math. Anal.* **42**, 539–567 (2010).

[43] C. L. Epstein and R. Mazzeo, Wright-Fisher diffusion in one dimension. *SIAM J. Math. Anal.* **42**, 568–608 (2010).

[44] A. Wagner, *Robustness and evolvability in living systems.* Princeton University Press, 2005.

[45] F. Heylighen. The science of self-organization and adaptivity. In L. D. Kiel (ed.), *The encyclopedia of life support systems.* EOLSS Publishers, 2003.

[46] T.-D. Lee and C.-N. Yang, Statistical theory of equations of state and phase transitions. II. Lattice gas and Ising model. *Phys. Rev.* **87**, 410 (1952).

[47] S. A. Kauffman. *The origins of order: Self-organization and selection in evolution.* Oxford University Press, 1993.

[48] M. Aldana, S. Coppersmith, and Leo P. Kadanoff, Boolean dynamics with random couplings. In E. Kaplan, J. E. Marsden, and K. R. Sreenivasan (eds.), *Perspectives and problems in nonlinear science.* Springer, 2003.

[49] G. P. Wagner and Lee Altenberg, Perspective: Complex adaptations and the evolution of evolvability. *Evolution* **50**, 967–976 (1996).
